# Prevalence, trends, and distribution of hepatitis C virus among the general population in sub‐Saharan Africa: A systematic review and meta‐analysis

**DOI:** 10.1111/liv.16102

**Published:** 2024-09-13

**Authors:** Getahun Molla Kassa, Josephine G. Walker, Tesfa Sewunet Alamneh, Melaku Tileku Tamiru, Sandra Bivegete, Aynishet Adane, Wondwossen Amogne, John F. Dillon, Peter Vickerman, Emebet Dagne, Elias Ali Yesuf, Matthew Hickman, Clare E. French, Aaron G. Lim, the DESTINE NIHR Global Health Research Group, John F. Dillon, Wondwossen Amogne, Peter Vickerman, Matthew Hickman, Ora Paltiel, Dawit Wolday, Aynishet Adane, Saro Abdella, Zenahbezu Abay, Workagegnehu Hailu, Tadesse Awoke, Emebet Dagne, Elias Ali Yesuf, Josephine G. Walker, Aaron G. Lim, Clare E. French, Andaragachew Mulu, Melaku Tileku Tamiru, Atsbeha Gebreegziabxier Weldemariam, Christie Cabral, Obsie Baissa, Elizabeth Speakman, Andrew Radley, Amy Malaguti, Sarah K. Inglis, Meseret Yohannes, Bruktait Taddele, Hagos Abraha, Mengistu Erkie, Tesfa Sewunet Alamneh, Getahun Molla Kassa

**Affiliations:** ^1^ Population Health Sciences, Bristol Medical School University of Bristol Bristol UK; ^2^ Department of Epidemiology and Biostatistics, Institute of Public Health, College of Medicine and Health Sciences University of Gondar Gondar Ethiopia; ^3^ Division of Molecular and Clinical Medicine, School of Medicine University of Dundee Dundee UK; ^4^ Department of Pharmacology and Clinical Pharmacy, School of Pharmacy, College of Health Sciences Addis Ababa University Addis Ababa Ethiopia; ^5^ Department of Internal Medicine, School of Medicine, College of Medicine and Health Sciences University of Gondar Gondar Ethiopia; ^6^ Department of Internal Medicine, School of Medicine, College of Health Sciences Addis Ababa University Addis Ababa Ethiopia; ^7^ Department of Internal Medicine, Institute of Health Jimma University Jimma Ethiopia; ^8^ Department of Health Policy and Management, Institute of Health Jimma University Jimma Ethiopia; ^9^ NIHR Health Protection Research Unit in Behavioural Science and Evaluation University of Bristol Bristol UK

**Keywords:** general population, HCV, seroprevalence, SSA, viraemic prevalence

## Abstract

**Background and Aims:**

Although the evidence is uncertain, existing estimates for hepatitis C virus (HCV) in sub‐Saharan Africa (SSA) indicate a high burden. We estimated HCV seroprevalence and viraemic prevalence among the general population in SSA.

**Methods:**

We searched Medline, Embase, Web of Science, APA PsycINFO, and World Health Organization Africa Index Medicus for community‐based studies. Study quality was assessed using the Joanna Briggs Institute critical appraisal tool, and heterogeneity using the index of heterogeneity (*I* ^2^). Two approaches were deployed. First, we used random‐effects meta‐analysis to pool prevalence. Second, to derive representative estimates, we weighted each country's HCV seroprevalence using 2021 United Nations country population sizes.

**Results:**

We synthesized 130 studies. Overall, SSA HCV seroprevalence from the random‐effects model was 4.17% (95% confidence interval [CI]: 3.71–4.66, *I* ^2^ = 99.30%). There were no differences between males (4.31%) and females (4.03%). Seroprevalence was 2.25%, 3.31%, and 16.23% for ages ≤20, 21–64, and ≥65 years, respectively, and was higher in rural (6.63%) versus urban (2.93%). There was indication of decrement overtime from 5.74% to 4.35% to 3.03% in the years 1984–2000, 2001–2014, and 2015–2023, respectively. The weighted overall SSA HCV seroprevalence was estimated to be 2.30% (95% CI: 1.59–3.00) with regional variation: Africa‐Southern (.79%), Africa‐Central (1.47%), Africa‐Eastern (2.71%), and Africa‐Western (2.88%). HCV viremia among HCV seropositives was 54.77% (95% CI: 47.80–61.66).

**Conclusions:**

HCV seroprevalence in SSA remains high. Populations aged ≥65 years, rural communities, Africa‐Western, and some countries in Africa‐Central and Africa‐Eastern appear disproportionately affected. These results underline the need for governmental commitment to achieve the 2030 global HCV elimination targets.

**Abbreviations**
CI: confidence interval
ELSA/EIA: Enzyme‐Linked Immunosorbent Assay/Enzyme Immune Assay
HCV: hepatitis C virus
*I* ^2^
: index of heterogeneity statistics
JBI: Joanna Briggs Institute
SSA: sub‐Saharan Africa
WHO: World Health Organization

**Key points**

- This review revealed the most comprehensive and up‐to‐date evidence on hepatitis C virus (HCV) epidemiology among the general population in sub‐Saharan Africa (SSA).
- We estimated the overall SSA HCV seroprevalence of 4.17% using random‐effects meta‐analysis and a population size weighted seroprevalence of 2.30%.
- We estimated HCV chronic infection of 54.77% among HCV antibody positives.
- We found significant variations in HCV prevalence across subregions, countries, urban/rural residence, age groups, and signs of HCV seroprevalence decrement over time.
- Findings from this review will help to prioritize specific segments of the general population for targeted community‐based HCV surveillance and interventions, such as community‐based screening.

## 1 INTRODUCTION

Chronic hepatitis c virus (HCV) infection is a leading cause of liver cirrhosis, liver cancer, liver transplantation, and related deaths worldwide.^1^, ^2^, ^3^ In 2019, the World Health Organization (WHO) estimated that 58 million people are living with chronic HCV infection globally.^4^

Available data indicate that sub‐Saharan Africa (SSA) carries a substantial burden of HCV. According to WHO estimates in 2019, the WHO African region ranks third in HCV prevalence, following Eastern Mediterranean and European regions.^4^ The Polaris Observatory Group estimated 7.8 million (.7%) chronic HCV infections in the WHO African region in 2020 of which .8 million (10.0%) were diagnosed and 30 700 (.4%) received treatment.^5^ In the Polaris study, the highest viremia was estimated for Africa‐Western (.8%) and Africa‐Eastern (.7%).^5^ These findings indicate that SSA is off track to meet the global HCV elimination targets of 90% diagnosis, 80% treatment, and a 65% reduction in mortality by 2030.^6^

Less than a decade away from these elimination targets, SSA still lacks robust epidemiological data for the general population.^7^ Existing systematic reviews and meta‐analysis are either outdated or suffer from various limitations such as over‐representation of data from African‐Northern countries like Egypt; employing restrictive search strategies; including populations with different risks of HCV transmission than the general population; generating estimates using data from few countries; or searching few databases.^8^, ^9^, ^10^, ^11^, ^12^, ^13^, ^14^

Comprehensive data on the prevalence and distribution of HCV in SSA's general population are crucial for informing policymakers and for devising and working towards effective elimination strategies. This review aimed to estimate the overall HCV prevalence in the general population of SSA, with further stratification by geographical regions, demographic factors, and calendar period.

## 2 METHODS

The protocol for this systematic review and meta‐analysis has been published in PROSPERO under the registration number CRD42023373986 and is accessible at https://www.crd.york.ac.uk/prospero/display_record.php?RecordID=373986.

### 2.1 Search strategy

We developed a comprehensive search strategy using subject headings and keywords. The search strategy covered four main domains: Epidemiology, Risk Factors, HCV, and Africa. We searched Ovid Medline, Embase via Ovid, APA PsycINFO via Ovid, Web of Science, and WHO Africa Index Medicus on October 10 and 11, 2022 and updated on 20 December 2023. Additionally, we manually searched reference lists of relevant published systematic reviews and meta‐analysis. There were no publication date, language, or sample size restrictions (see Table S1).

### 2.2 Eligibility criteria

We included cross‐sectional, cohort, and case–control studies reporting HCV seroprevalence, viraemic prevalence, and risk factors among the general population. This covered all 48 countries listed by the World Bank Group under SSA countries, as well as Djibouti and two French dependencies (Reunion and Mayotte).^15^ The term ‘general population’ refers to any community‐based survey and includes house‐to‐house surveys and samples from portions of the communities such as specific age/sex groups, or populations without reference to any low or high‐risk characteristics for HCV transmission such as students. We excluded studies conducted among high‐risk or key populations for HCV transmission or acquisition, as well as studies conducted among subjects in healthcare facilities, comorbid/chronic illnesses, factory workers, and migrants. Studies lacking data either on HCV prevalence or risk factors were also excluded (see Table S2).

### 2.3 Study selection

Database search hits were exported and deduplicated using EndNote‐20. Titles and abstracts were double‐screened in Rayyan^16^ followed by dual full‐text screening using Covidence.^17^ Discrepancies were resolved through discussion.

### 2.4 Data extraction and quality assessment

All data were extracted in duplicate blindly. Where multiple reports existed from the same dataset (e.g. conference abstracts and full reports), we extracted data from the most comprehensive or most recent publication, cross‐referencing with associated reports as needed. Extracted data included study area, study design, populations, sampling techniques, study start and end years, sample size, HCV diagnostic methods, demographic characteristics, and the number of HCV‐tested and HCV positives for both seroprevalence and viraemic prevalence.

We employed the Joanna Briggs Institute (JBI) critical appraisal tool for prevalence studies to assess the quality of the included articles.^18^ This tool comprises nine questions that evaluate the study's internal and external validity, with possible answers being ‘Yes’, ‘No’, ‘Unclear’, or ‘Not applicable’. Since we expected all included articles to cover these nine domains, the ‘Not Applicable’ classification was not used (see Table S3). Finally, we summed the number of yeses for each study and grouped those articles that scored seven yeses as high quality, six or seven yeses as moderate quality, and below six yeses as poor quality.

### 2.5 Age, calendar years, and SSA subregional classifications

We categorized ages into: ≤20 (0 to 20), 21–64, and ≥65 years (see Table S4). Study years were categorized into three calendar periods: pre‐2001, 2001–2014, and 2015–2023 based on the introduction of pegylated‐interferon and direct‐acting antivirals treatment globally.^19^ We grouped SSA countries into Africa‐Eastern, Africa‐Western, Africa‐Southern, and Africa‐Central based on WHO geography and actual geographical location (see Table S5).

### 2.6 Data analysis/synthesis

Extracted data were cleaned, recoded and analysed in STATA/MP 18.^20^ We summarized the characteristics of the included studies with respect to geographical characteristics, study design, HCV testing methods, and calendar periods.

We used two analysis approaches in this review. First, we used random‐effects meta‐analysis to estimate pooled overall, subregional, and subgroup HCV seroprevalence and viraemic prevalence with sensitivity analyses. Second, we estimated the overall and subregional HCV seroprevalences weighted according to the population size of each country. These two approaches are described in more detail below.

### 2.7 Overall and subgroup seroprevalence and viraemic prevalence estimates from random‐effects meta‐analyses

A random‐effects meta‐analysis model was executed to generate subgroup prevalence estimates, thereby accounting for within‐study and between‐study variations. Pooled prevalence estimates were calculated using the *metaprop* STATA command^21^ and variance was stabilized using the Freeman‐Tukey Double Arcsine Transformation.^22^

We estimated overall and subregional pooled prevalence. To enhance the robustness of our findings, we undertook several sensitivity analyses: (a) incorporating the JBI critical appraisal assessments; (b) performing an influential studies analysis, by excluding studies identified as outliers; and (c) restricting to studies that used Enzyme‐Linked Immunosorbent Assay/Enzyme Immune Assay (ELISA/EIA) diagnosis kits. We also conducted subgroup analysis by country, sex, age, urban/rural residency, and calendar periods. We report prevalence as point estimates accompanied by 95% confidence intervals (CI).

Heterogeneity of studies in the meta‐analysis was assessed using the Cochran *Q* test and the Index of heterogeneity (*I* ^2^) statistic.^23^

### 2.8 Weighted overall and subregional seroprevalence estimates

We selected the best available estimates for each country from the included studies. For countries without data, we imputed data from the WHO African region hepatitis scorecard^24^ or the neighbouring/nearest country (see Table S6). After obtaining the best estimate for each country, we estimated overall and subregional seroprevalence using country‐level estimates weighted by the 2021 United Nations population estimates of country's population size.^25^ It was not possible to weight overall viraemic prevalence and seroprevalence by age, sex, residency, or calendar period due to the lack of population size estimates stratified by those factors in most of the countries.

## 3 RESULTS

### 3.1 Characteristics of included studies

**FIGURE 1:**
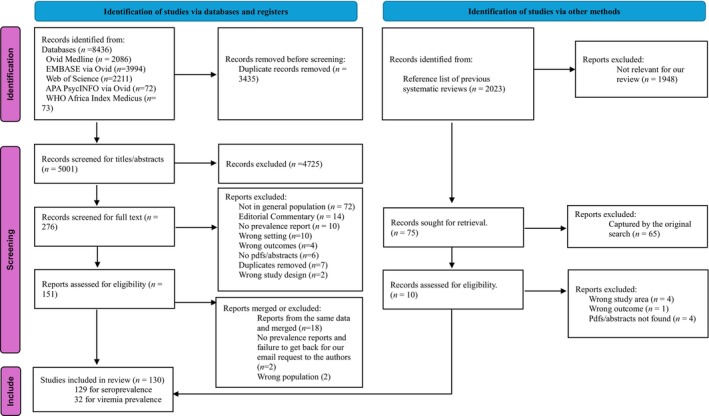
Preferred Reporting Items for Systematic Reviews and Meta Analysis 2020 flow diagram which included databases searches and other sources.

Our search strategy identified 10 459 records. Following deduplication, titles/abstracts screening, and full text screening, 261 records were assessed for eligibility and 130 studies were included (129 for seroprevalence and 32 for viremia) from 33 countries which represent over 85% of the SSA population (see Figure 1). There were no eligible studies for Comoros, Djibouti, Mozambique, Mauritius, Cabo Verde, Cote d'Ivoire, Liberia, Mauritania, Niger, Togo, Angola, Chad, São Tomé and Príncipe, Botswana, Eswatini, Lesotho, Namibia, and Reunion. From the total 1 920 287 sample size in the 129 seroprevalence studies, there were 1 683 802 (87.68%) individuals with HCV antibody test result. Of those, 1 584 929 were HCV antibody negative, 94 826 were HCV antibody positive, and 4047 had indeterminant results. We removed all indeterminant results from our analysis.

Of the included 130 studies, 97 reported HCV seroprevalence only, 32 reported both HCV seroprevalence and viraemic prevalence, and one reported only HCV viraemic prevalence. Nearly all studies (98.54%, *n* = 128/130) were cross‐sectional, and two were retrospective record reviews of community‐based mass screening survey data. Three studies reported HCV seroprevalence in two or more countries and we extracted data separately for each country, resulting in 137 datasets (136 for seroprevalence and 32 for viraemic prevalence). Of those datasets, 50 (36.50%) were in Africa‐Western, 49 (35.77%) were in Africa‐Eastern, 32 (23.36%) were in Africa‐Central, and 6 (4.38%) were in Africa‐Southern regions. The countries contributing the most datasets were: Nigeria (*n* = 29; 21.17%), Cameroon (*n* = 18; 13.14%), Ethiopia (*n* = 11;8.03%), and Tanzania (*n* = 9; 6.57%). From 136 seroprevalence datasets, 99 (72.79%) used ELISA/EIA to test HCV antibody, 25 (18.38%) used rapid diagnostic kits, and 12 (8.82%) reported antibody tests but didn't specify the type (see Table S11 for details).

In the JBI quality assessment, of 137 datasets, 53 were grouped under high quality, 41 under moderate quality, and 43 under poor quality (see Figure S2).

### 3.2 Overall and subgroup HCV seroprevalence from the random‐effects meta‐analysis

**FIGURE 2:**
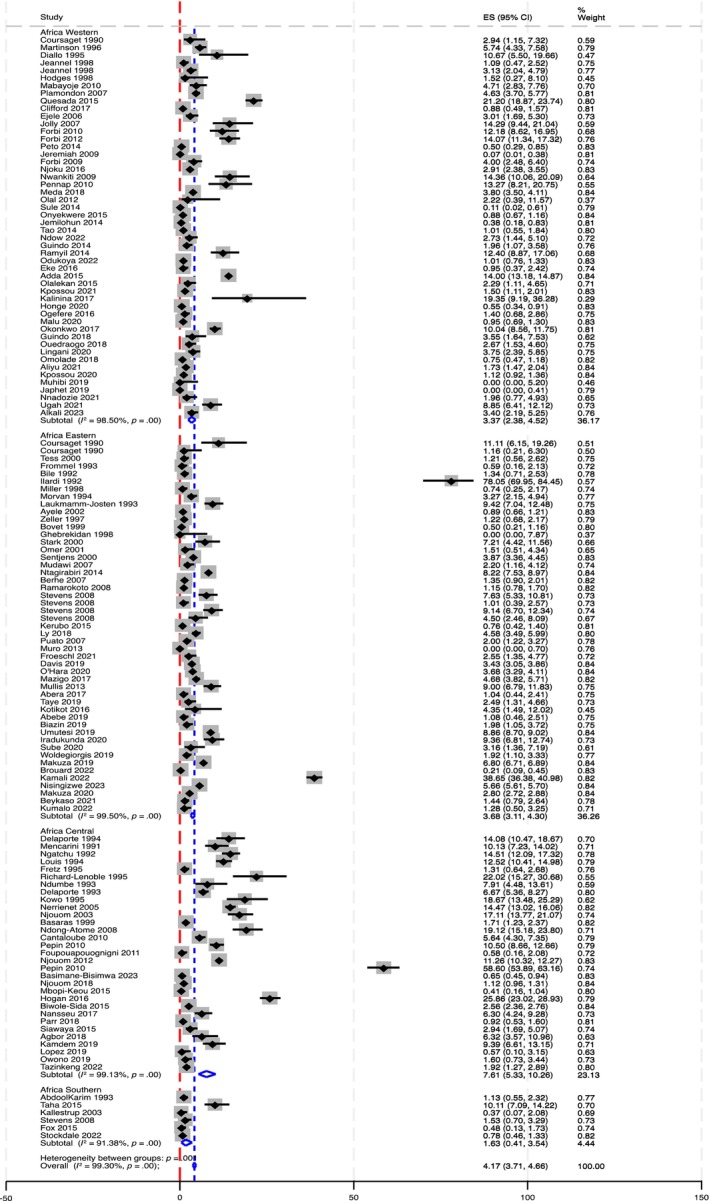
Forest plot for HCV seroprevalence using all included studies stratified by subregions of sub‐Saharan Africa.

**FIGURE 3:**
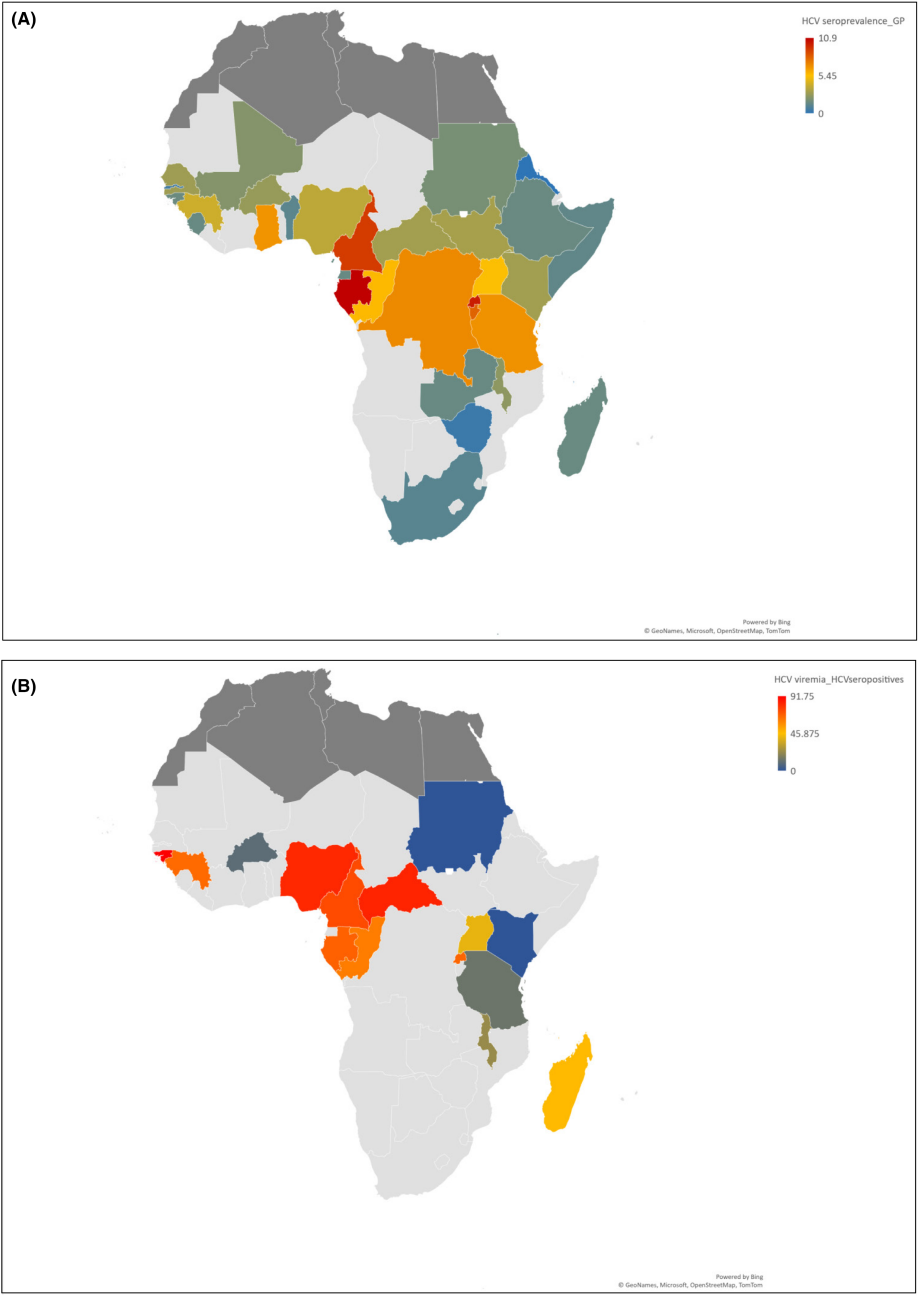
Country‐level geographical distribution of HCV seroprevalence (A) and viraemic prevalence among HCV seropositive (B) among the general population in sub‐Saharan Africa (grey areas are countries without data and dark areas are Africa‐Northern region).

There was substantial heterogeneity (*I* ^2^ >90%) throughout the analysis. Overall, across all included studies HCV seroprevalence in SSA was 4.17% (95% CI: 3.71–4.66, *I* ^2^ = 99.30%) with subregional variations: 7.61% (95% CI: 5.33–10.26) in Africa‐Central, 3.68% (95% CI: 3.11–4.30) in Africa‐Eastern, 3.37% (95% CI: 2.38–4.52) in Africa‐Western, and 1.63% (95% CI: .41–3.54) in Africa‐Southern (see Figure 2). The countries Gabon (10.90%, 95% CI: 6.47–16.29), Rwanda (10.05%, 95% CI: 7.92–12.40), Cameroon (9.19%, 95% CI: 5.82–13.21), and Burundi (8.05%, 95% CI: 7.34–8.78) had the highest country‐level HCV seroprevalences (see Figure 3A, Table S7).

Three studies from Tanzania,^26^ Cameroon,^27^ and Rwanda^28^ reported substantially higher seroprevalence (see Figure 2). We anticipated that these studies might skew the pooled HCV seroprevalence. To test this, we performed influential study analysis by excluding them which gave a pooled seroprevalence of 3.61% (95% CI: 3.20–4.05) for the whole SSA, and 2.81% (95% CI: 2.33–3.33) for Africa‐Eastern, 6.57% (95% CI: 4.65–8.79) for Africa‐Central, 2.67% (95% CI: .99–5.08) for Tanzania, 6.29% (95% CI: 4.61–8.22) for Rwanda, and 7.19% (95% CI: 4.74–10.11) for Cameroon. Heterogeneity remained unchanged (*I* ^2^ >94% in all the estimates).

From the 136 HCV seroprevalence datasets, 83 reported seroprevalence by sex, 82 by different age categories, and 95 by urban/rural residency. In SSA, the groups with the highest HCV seroprevalence were those aged ≥65 years (16.23%, 95% CI: 13.24–19.44) and those residing in rural areas (6.63%, 95% CI: 5.18–8.23). It was 4.31% (95% CI: 3.61–5.07) among males and 4.03% (95% CI: 3.45–4.66) among females. In the four SSA subregions, HCV seroprevalence among the rural population was 2.59% (95% CI: .00–9.53), 5.70% (95% CI: 3.29–8.67), 6.54% (95% CI: 4.63–8.75), and 8.95% (95% CI: 5.50–13.13) in Africa‐Southern, Africa‐Eastern, Africa‐Western, and Africa‐Central, respectively (see Table 1).

**TABLE 1:** HCV seroprevalence among the general population in sub‐Saharan Africa by sex, age, and residency from the random‐effects meta‐analysis (using *N* = 136 datasets).

Variables	Group	Regions of SSA					
			Eastern	Western	Central	Southern	SSA
Sex	Male	P (95% CI)	2.34 (1.66–3.12)^a^	3.04 (1.95–4.35)^b^	11.28 (6.26–17.51)^a^	1.04 (.00–3.15)^d^	4.31 (3.61–5.07)^a^
		*k*	25	27	21	2	75
		*n*/*N*	28 181/466350	576/19470	909/16300	3/215	29 669/502335
	Female	P (95% CI)	2.75 (2.07–3.52)^a^	3.41 (1.97–5.18)^a^	9.03 (5.20–13.77)^a^	.64 (.18–1.30)^d^	4.03 (3.45–4.66)^a^
		*k*	30	28	21	3	82
		*n*/*N*	57 689/981918	846/22594	975/18079	7/989	59 517/1023580
Age in years	≤20	P (95% CI)	2.85 (1.54–4.49)^a^	2.06 (.10–5.49)^a^	1.62 (.25–3.56)^a^	—^d^	2.25 (1.19–3.56)^a^
		*k*	19	16	14	0	49
		*n*/*N*	556/56 011	1122/17 716	171/8422	—/—	1849/82 149
	21–64	P (95% CI)	2.19 (1.70–2.73)^a^	4.20 (2.43–6.41)^a^	6.28 (4.52–8.30)^a^	.86 (.51–1.29)^d^	3.31 (2.87–3.79)^a^
		*k*	28	23	19	2	72
		*n*/*N*	45 071/1 113 729	1075/31 481	1392/46 414	20/2278	47 558/1 193 902
	>64	P (95% CI)	11.92 (8.15–16.21)^a^	7.54 (3.09–13.40)^c^	28.07 (17.08–40.50)^a^	—^d^	16.23 (13.24–19.44)^a^
		*k*	13	6	11	0	30
		*n*/*N*	38 883/171 141	101/1377	1052/4028	—/—	40 036/176 546
Residency	Urban	P (95% CI)	2.00 (1.23–2.94)^a^	2.41 (1.32–3.80)^a^	8.87 (3.80–15.76)^a^	1.03 (.45–1.81)^d^	2.93 (2.19–3.77)^a^
		*k*	18	24	8	3	53
		*n*/*N*	1697/90 903	733/28 738	1283/35 503	22/2230	3735/157 374
	Rural	P (95% CI)	5.70 (3.29–8.67)^a^	6.54 (4.63–8.75)^b^	8.95 (5.50–13.13)^a^	2.59 (.00–9.53)^d^	6.63 (5.18–8.23)^a^
		*k*	22	12	16	3	53
		*n*/*N*	3458/104 706	738/14 317	964/16 930	33/987	5193/136 940

*Note*: All Q statistic *p*‐values were <.01. There were no HCV seroprevalence data for the Africa‐Southern region for the age group ≤20 and ≥65 years.

Abbreviations: CI, Confidence Interval; *I* ^2^, Index of Heterogeneity; *k*, number of pooled studies; *n*, number of HCV seropositive; *N*, number of tested for HCV serology; P, pooled seroprevalence; SSA, Sub‐Saharan Africa.

^a^ *I* ^2^ >95%.

^b^ *I* ^2^ 90%–95%.

^c^ *I* ^2^ <90%.

^d^ *I* ^2^ not applicable.

**FIGURE 4:**
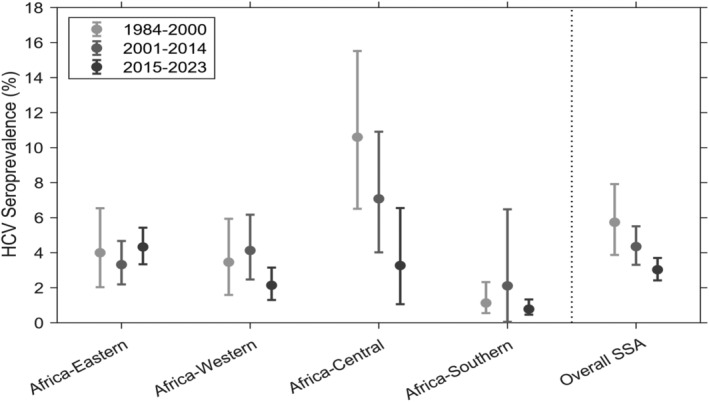
Seroprevalence of hepatitis C virus over‐time among the general population in sub‐Saharan Africa and its subregions.

Overall HCV seroprevalence appeared to decline across calendar periods and showed substantial heterogeneity (*I* ^2^ >95%). It was 5.74% (95% CI: 3.87–7.82), 4.35% (95% CI: 3.31–5.51), and 3.03% (95% CI: 2.42–3.70) in the years 1984–2000, 2001–2014, and 2015–2023, respectively (see Figure 4 and Figure S1). In the same chronological order, decrement was also observed in Africa‐Central but not in other subregions. The list of included countries varies across each calendar period. In countries with better data across the calendar periods, we observed a stable trend in Burkina Faso and Ethiopia and decrement in Cameroon, Nigeria, and Tanzania (see Table S9).

### 3.3 Weighted overall and subregional HCV seroprevalences

The overall weighted HCV seroprevalence was 2.30% (95% CI: 1.59–3.00), with regional variation from .79% (95% CI: .53–1.05) in Africa‐Southern to 2.88% (95% CI: 1.12–4.63) in Africa‐Western (see Table 2).

**TABLE 2:** Weighed HCV seroprevalence in Sub‐Saharan Africa and its subregions.

Geographical regions	Weighed HCV seroprevalence (95% CI)
Sub‐Saharan Africa	2.30% (1.59–3.00)
Africa‐Western	2.88% (1.12–4.63)
Africa‐Eastern	2.71% (1.81–3.60)
Africa‐Central	1.47% (.25–2.70)
Africa‐Southern	.79% (.53–1.05)

### 3.4 HCV viraemic prevalence from random‐effects meta‐analyses

We estimated HCV viraemic prevalence using 32 studies from 17 countries covering 55% of the SSA population. Among HCV seropositives, viraemic prevalence was 54.77% (95% CI: 47.80–61.66), varying from 41.54% (95% CI: 32.35–51.02) in Africa‐Eastern to 71.10% (95% CI: 49.89–88.81) in Africa‐Central (see Figure 3B). The pooled HCV viraemic prevalence among the general population using 32 studies from 17 countries was 2.64% (95% CI: 2.00–3.52). It ranged from 1.56% (95% CI: .79–2.31) in Africa‐Eastern to 5.89% (95% CI: 2.93–9.78) in Africa‐Central (see Table S10).

## 4 DISCUSSION

The overall weighted HCV seroprevalence in SSA was 2.30%. Geographical variations were observed with the highest seroprevalence in Africa‐Western (2.88%) followed by Africa‐Eastern (2.71%), Africa‐Central (1.47%), and Africa‐Southern (.79%), respectively. At the country level Gabon (10.90%), Rwanda (10.05%), Cameroon (9.19%), and Burundi (8.05%) carried the greatest burden. Although the seroprevalence in Cameroon and Rwanda lowered when excluding outlier reports by Pepin 2010^27^ and Kamali 2022.^28^

Our overall weighted HCV seroprevalence estimate was much lower than previously published reviews.^8^, ^9^, ^10^, ^11^, ^12^, ^13^, ^14^ This may be explained by methodological differences between our review and previously published reviews. We calculated the overall weighted estimate from country‐level HCV seroprevalance estimates weighted by 2021 UN population sizes and we believe this will be the best estimate for the overall SSA and its subregions. However, our 4.17% seroprevalence estimate (*I* ^2^ = 99.30%) from the random‐effects meta‐analysis using 129 studies from 33 out of 51 SSA countries covering 85% of the SSA population is also lower than the recent systematic review report of 7.1% by Salari et al. which was for the whole Africa and used data from only 16 studies, 12 of which studies were from Africa‐Northern countries (10 in Egypt).^8^ Our estimate was also lower than the Mora et al. estimate of 5.4% using 68 studies,^12^ Rao et al. estimate of 6.9% using 88 studies,^13^ and Karoney and Siika, estimates of 5.3% using 49 studies.^14^ The Mora et al. estimate used data published from 2000 to 2013 and is in line with our estimate for the years 2001–2014. The main difference between our estimate and the Mora et al. estimate was the definition of general population. They defined general population as pregnant women, household members, adults, outpatients, healthy children, and infants. They also stated that pregnant women have lower HCV infection as compared to the general population, which might underestimate the seroprevalence of the general population. The Rao et al. includes data from 2002 to 2014 which is higher than our estimate for the year 2001–2014. However, their eligibility criteria also differed as they included samples from the community, students, and inpatients and outpatients who did not have a history of multiple blood transfusion or seeking care for non‐hepatic illness were included, with a chance that their symptoms might be from chronic HCV infection. The higher estimate from the random‐effects meta‐analysis as compared to the weighted estimate in this review is due to the higher weight given by the random‐effects model to studies with higher prevalence but smaller sample size and studies from higher HCV prevalence countries with lower population sizes. The lower seroprevalence in our review compared to older estimates might be related to improvements in medical injections and blood transfusion safety^29^, ^30^; and/or progressive loss of the higher seroprevalence population due to ageing and liver related mortality.

There is an indication of HCV seroprevalence decrement over time, being 5.74% during the earlier calendar period (1984–2000) and 3.03% during the most recent calendar period (2015–2023). However, more detailed sub‐regional investigation revealed that this decrement was only observed in Africa‐Central. This overtime decrement might be related to improvements in the control of iatrogenic infection transmission such as increased safety of blood transfusions^29^ and medical procedures including banning the reuse of injecting equipment.^30^ Meanwhile, the contribution of increased diagnosis and treatment, another potential reason for this over‐time seroprevalence change, might be minimal since HCV diagnosis and treatment has been very low in SSA.^4^ The decrement might not be precise since the list of countries included in each calendar period is different and studies in the recent periods might have used more specific testing methods. These considerations make it challenging to interpret the decrement at face value and so warrants further investigation.

HCV seroprevalence varied by age. Those aged ≥65 years had four‐times (16.23%) higher seroprevalence than those aged 21–64 years (3.31%) and five‐times higher than those aged ≤20 years (2.25%). In line with this, Mohd Hanafiah et al. reported a trend of increased HCV seroprevalence with increasing age in SSA, with a peak at 55–64 years.^31^ Our estimate for those aged ≤20 years is in line with a systematic review by Melikoki et al. which reported a 3.0% prevalence.^32^ However, the estimate of Melikoki et al. was for the whole of Africa based on 13 studies (6/13 were from Egypt) published from 1992 to 2016. This higher prevalence among the older population might reflect the presence of historical iatrogenic transmissions using contaminated healthcare‐related injections/procedures.^33^ However, the higher seroprevalence in the older age group should be interpreted cautiously since it is estimated from a subset of the included data and may not represent the general older population.

We found that rural populations had over twice the seroprevalence of urban populations (6.63% vs. 2.93%). The WHO 2021 global progress report on HIV, viral hepatitis, and sexually transmitted infections also reported the rural population are more affected than urban populations.^4^ There might be some behavioural/social factors that increase vulnerability of the rural population for HCV infection such as poor education and more practicing of tribal scarring using contaminated materials.

There were 32 included studies from 17 countries (representing 55% of SSA population) reporting viraemic prevalence. Among HCV seropositives, viraemic prevalence appears steady across the overall (54.77%), high‐quality studies (51.86%), and across calendar periods (55.68% for the years 2001–2014 and 59.04% for the years 2015–2023). Our overall, Africa‐Western, and Africa‐Central estimates are in line with the WHO chronic HCV estimate of 70%,^34^ while the Africa‐Eastern estimates are lower. The relatively lower HCV viraemic prevalence among HCV seropositives and variability across the subregions of SSA might be related to: the validity of different testing methods; higher natural viral clearance due to HCV genotype or viral diversity circulated; differences in host factors such as age, sex, race, genetics, and immune response; and other epidemiological factors.^35^, ^36^ SSA populations are generally young^25^ and there is evidence that showed higher natural clearance of HCV viremiais in younger age groups.^36^

Overall, we estimated viremia prevalence of 2.64% among the general population. It was lower (1.67%) based on high‐quality studies only. Regionally, African‐Central (5.89%) had the highest viremia followed by the Western (2.06%) and Eastern (1.56%). Our overall viremia estimate is higher than that of the WHO 2021 global progress estimate of .8% for the year 2019^4^ and the Polaris Observatory group estimates of .8% and .7% for the WHO African region for 2015 and 2020, respectively.^5^ However, our estimate for high‐quality studies in the period 2015–2023 (1.08%) is in line with those two estimates. Both the WHO and the Polaris Observatory group estimated viraemic prevalence using empirical and programmatic data from a literature review of peer‐reviewed studies, grey literature, non‐indexed publications, government reports, and Delphi process of personal communication with experts, and applying a Markov model to forecast HCV prevalence. The Polaris Observatory group estimates use the Global Burden of Disease region to calculate population‐weighted regional averages. Our viraemic estimate may not be representative of SSA as a whole as we found few studies reporting these data, and these studies originated from just 17 countries.

We conducted a comprehensive and rigorous systematic review of published data, and to our knowledge, this provides the most up‐to‐date and representative estimate of HCV seroprevalence in SSA. However, there are several limitations pertaining to the available data. First, as might be anticipated, there was high heterogeneity which couldn't be explained by sensitivity and subgroup analysis considering study quality, geography, sex, age, and calendar period. Other behavioural and social risk factors and methodological differences across the included studies may contribute to this heterogeneity. Second, Africa‐Southern was underrepresented (six included studies from 4/10 countries) and there were also countries with one or very few included studies with zero or excessively high prevalence which may not appropriately represent the general population (for example, three studies reported HCV seroprevalence >30%, see Figure 2). Third, included studies used various diagnostic tests with different sensitivity and specificity. This performance variability could explain the wide variability seen in prevalence. Usually, HCV is tested in two stages: the first test will be very sensitive, followed by a second confirmatory test. Fourth, since the number of included studies for viremia are few, we didn't estimate a weighted viremia prevalence. Finally, though we comprehensively searched for published studies, we didn't search the grey literature. However, we did use the WHO African region HCV scorecard HCV seroprevalence estimates for countries with missing estimates when generating our best estimate weighted by population size.

In conclusion, HCV prevalence in SSA is high, particularly among older individuals, rural communities, Africa‐Western, and some countries in the Africa‐Central and Africa‐Eastern regions. This high prevalence with disproportionally affected population groups, combined with the lack of data for HCV in the general populations in some SSA countries, stresses the need for systematic interventions to address these problems. The data presented in this review are important for policy to map the burden and to plan and allocate resources. We observed a potential decline in recent years in HCV prevalence among the general populations, which may hint that countries can eliminate HCV, if they implement systematic interventions for the diagnosis, treatment, and prevention of HCV infections. These results underline the need for high governmental commitment to achieve the 2030 global HCV elimination targets in SSA countries.

## AUTHOR CONTRIBUTIONS

GMK conceptualized the study with MH, AGL, JW, and CEF. GMK developed the protocol with support from MH, AGL, JW, CEF, and PV. GMK developed the search strategy with support from AGL, JW, and MH. GMK conducted the database searching. GMK screened titles/abstracts and full texts and double screened by AGL, JW, CEF, TSA, MTT, and SB. GMK extracted the data and double extracted by TSA, MTT, and SB. GMK analysed and interpreted the data with support from CEF, AGL, JW, and MH. GMK drafted the original manuscript with support from AGL, JW, CEF, and MH. All authors edited, commented, and revised the manuscript. All authors approved the final version of the manuscript.

## FUNDING INFORMATION

This research was funded by the NIHR (NIHR133208) using UK international development funding from the UK Government to support global health research. The views expressed in this publication are those of the author(s) and not necessarily those of the NIHR or the UK Department of Health and Social Care.

## CONFLICT OF INTEREST STATEMENT

The authors do not have any disclosures to report.

## Supporting information


Data S1.

## Data Availability

The source of all underline data of this systematic review and meta‐analysis are available in the supplementary file and for additional information the lead author can be contacted.
