# Supplementary material for: Prevalence, trends, and distribution of hepatitis C virus among the general population in sub‐Saharan Africa: A systematic review and meta‐analysis
Source: Liver Int. 2024 Sep 13;44(12):3238–49. doi: 10.1111/liv.16102 (PMC11586889; doi:10.1111/liv.16102)
Supplement: Supplementary file 1 — Data S1. [file LIV-44-3238-s001.docx]

**Prevalence, Trends, and Distribution of Hepatitis C Virus among the General Population in Sub-Saharan Africa: A Systematic Review and Meta-Analysis**

**Supplementary File**

Getahun Molla Kassa^1,2*^, Josephine G Walker^1^, Tesfa Sewunet Alamneh^1,2^, Melaku Tileku Tamiru^3,4^, Sandra Bivegete^1^, Aynishet Adane^5^, Wondwossen Amogne^6^, John F Dillon^3^, Peter Vickerman^1^, Emebet Dagne^7^, Elias Ali Yesuf^8^, Matthew Hickman^1^, Clare E French^1,9^^, Aaron G Lim^1^^, on behalf of the DESTINE NIHR Global Health Research Group.

**Authors Affiliations**

^1^Population Health Sciences, Bristol Medical School, University of Bristol, Bristol, UK.

^2^Department of Epidemiology and Biostatistics, Institute of Public Health, College of Medicine and Health Sciences, University of Gondar, Gondar, Ethiopia.

^3^Division of Molecular and Clinical Medicine, School of Medicine, University of Dundee, Dundee, UK.

^4^Department of Pharmacology and Clinical Pharmacy, School of Pharmacy, College of Health Sciences, Addis Ababa University, Addis Ababa, Ethiopia.

^5^Department of Internal Medicine, School of Medicine, College of Medicine and Health Sciences, University of Gondar, Gondar, Ethiopia.

^6^Department of Internal Medicine, School of Medicine, College of Health Sciences, Addis Ababa University, Addis Ababa, Ethiopia.

^7^Department of Internal Medicine, Institute of Health, Jimma University, Jimma, Ethiopia.

^8^Department of Health Policy and Management, Institute of Health, Jimma University, Jimma, Ethiopia.

^9^NIHR Health Protection Research Unit in Behavioural Science and Evaluation at University of Bristol, Bristol, UK.

^Joint senior authors

*Corresponding Author

**Email**: [getahun.kassa@bristol.ac.uk](mailto:geteahun.kassa@bristol.ac.uk)

1. **Methods**
   1. **Search strategies**

The search strategy had four main concepts: Epidemiology, Risk Factors, Hepatitis C Virus, and Africa. It was constructed by looking at literature, databases, and published systematic reviews and meta-analyses on the topic. We entirely listed the potential subject headings and keywords in each search domain and combined them with different Boolean operators. Initial search was conducted in October 10 and 11, 2022 and updated on December 20, 2023 (see S**upplementary Table S1)**.

**Supplementary table S1:** Database search strategies

| Concepts | Ovid MEDLINE(R) <1946 to present>  Date run:10^th^ of October 2022. | Embase <1974 to 2022 October 10>  Via Ovid Platform | Web of Science  Date run: October 11, 2022. 15:13:43 BST | APA PsycInfo <1806 to October Week 1 2022>  Via Ovid Platform | Africa Index Medicus  Date run: October 11, 2022, 17:50 BST |
| --- | --- | --- | --- | --- | --- |
| 1. Epidemiology | exp Epidemiology/ OR exp Morbidity/ OR exp Prevalence/ OR exp Incidence/ OR Epidemiology.tw,kw. OR Prevalence*.tw,kw. OR incidence*.tw,kw. OR Attack Rate*.tw,kw. OR epidemic*.tw,kw. OR frequency.tw,kw. OR morbidity.tw,kw. OR outbreak*.tw,kw. OR endemic*.tw,kw. or rate.tw,kw. | exp Epidemiology/ OR exp Morbidity/ OR exp Prevalence/ OR exp Incidence/ OR Epidemiology.tw,kw. OR Prevalence*.tw,kw. OR incidence*.tw,kw. OR Attack Rate*.tw,kw. OR epidemic*.tw,kw. OR frequency.tw,kw. OR morbidity.tw,kw. OR outbreak*.tw,kw. OR endemic*.tw,kw. or rate.tw,kw. | Epidemiology (Topic) OR Morbidity (Topic) OR Prevalence* (Topic) OR incidence* (Topic) OR "Attack Rate*" (Topic) OR epidemic* (Topic) OR frequency (Topic) OR outbreak* (Topic) OR endemic* (Topic) OR rate (Topic) | exp epidemiology/ OR exp morbidity/ OR Epidemiology.tw. OR Prevalence*.tw. OR incidence*.tw. OR Attack Rate*.tw. OR epidemic*.tw. OR frequency.tw. OR morbidity.tw. OR outbreak*.tw. OR endemic*.tw. OR rate.tw. | Final search: (tw:((tw:((tw:(Epidemiology)) OR (tw:(Prevalence*)) OR (tw:(incidence*)) OR (tw:( “Attack Rate*”)) OR (tw:(epidemic*)) OR (tw:(frequency)) OR (tw:(morbidity)) OR (tw:(outbreak*)) OR (tw:(endemic*)) OR (tw:(rate)))) OR (tw:((tw:("Risk Factors*")) OR (tw:("Factor, Risk")) OR (tw:("Health Correlates")) OR (tw:("Associated Factor*")) OR (tw:(Predictor*)) OR (tw:(Determinant*)))))) AND (tw:((tw:("hepatitis c virus*")) OR (tw:("hepatitis c")) OR (tw:(HCV)) OR (tw:("hep c")) OR (tw:(hepacivirus*)) OR (tw:("Hepatitis C Virus Antibodies")) OR (tw:("HCV Antibodies")) OR (tw:("Anti?HCV Antibodies")) OR (tw:("Anti?Hepatitis C Virus Antibodies")) OR (tw:("Hepatitis C Antigen")) OR (tw:(Chronic Hepatitis C)))) |
| 1. Risk factors | exp Risk Factors/ OR Risk Factor*.tw,kw. OR Factor, Risk.tw,kw. OR Health Correlate*.tw,kw. OR Associated Factor*.tw,kw. OR Predictor*.tw,kw. OR Determinant*.tw,kw. | exp Risk Factors/ OR Risk Factor*.tw,kw. OR Factor, Risk.tw,kw. OR Health Correlate*.tw,kw. OR Associated Factor*.tw,kw. OR Predictor*.tw,kw. OR Determinant*.tw,kw. | "Risk Factor*" (Topic) OR "Factor, Risk" (Topic) OR "Health Correlates" (Topic) OR "Associated Factor*" (Topic) OR Predictor* (Topic) OR Determinant* (Topic) | exp Risk Factors/ OR Factor, Risk.tw. OR Risk Factor*.tw. OR Health Correlates.tw. OR Associated Factor*.tw. OR Predictor*.tw. OR Determinant*.tw. |  |
| 1. Hepatitis C Virus | exp Hepatitis C/ OR exp Hepacivirus/ OR exp Hepatitis C Antibodies/ OR exp Hepatitis C Antigens/ OR exp Hepatitis C, Chronic/ OR hepatitis c virus*.tw,kw. OR hepatitis c.tw,kw. OR HCV.tw,kw. OR hep C.tw,kw. OR hepacivirus*.tw,kw. OR Hepatitis C Virus Antibodies.tw,kw. OR HCV Antibodies.tw,kw. OR Anti?HCV Antibodies.tw,kw. OR Anti?Hepatitis C Virus Antibodies.tw,kw. OR Hepatitis C Antigen.tw,kw. OR Chronic Hepatitis C.tw,kw. | exp Hepatitis C/ OR exp Hepacivirus/ OR exp Hepatitis C Antibodies/ OR exp Hepatitis C Antigens/ OR exp Hepatitis C, Chronic/ OR hepatitis c virus*.tw,kw. OR hepatitis c.tw,kw. OR HCV.tw,kw. OR hep C.tw,kw. OR hepacivirus*.tw,kw. OR Hepatitis C Virus Antibodies.tw,kw. OR HCV Antibodies.tw,kw. OR Anti?HCV Antibodies.tw,kw. OR Anti?Hepatitis C Virus Antibodies.tw,kw. OR Hepatitis C Antigen.tw,kw. OR Chronic Hepatitis C.tw,kw. | "hepatitis c virus*" (Topic) OR "hepatitis c" (Topic) OR HCV (Topic) OR "hep c" (Topic) OR hepacivirus* (Topic) OR "Hepatitis C Virus Antibodies" (Topic) OR "HCV Antibodies" (Topic) OR "Anti?HCV Antibodies" (Topic) OR "Anti HCV Antibodies" (Topic) OR "Anti?Hepatitis C Virus Antibodies" (Topic) OR "Anti Hepatitis C Virus Antibodies" (Topic) OR "Hepatitis C Antigen" (Topic) OR "Chronic Hepatitis c" (Topic) | exp Hepatitis/ OR hepatitis c virus*.tw. OR hepatitis c.tw. OR HCV.tw. OR hep C.tw. OR hepacivirus*.tw. OR Hepatitis C Virus Antibodies.tw. OR HCV Antibodies.tw. OR Anti?HCV Antibodies.tw. OR Anti?Hepatitis C Virus Antibodies.tw. OR Hepatitis C Antigen.tw. OR Chronic Hepatitis C.tw. |  |
| 1. Africa | exp Africa/ or exp Africa, Eastern/ or exp Africa, Western/ or exp Africa, Southern/ or exp "Africa South of the Sahara"/ or exp Africa, Northern/ or exp Africa, Central/ OR Africa.tw,kw. OR sub?Sahara Africa.tw,kw. OR Algeria.tw,kw. OR Egypt.tw,kw. OR Libya.tw,kw. OR Morocco.tw,kw. OR Sudan.tw,kw. OR Tunisia.tw,kw. OR Burundi.tw,kw. OR Comoros.tw,kw. OR Djibouti.tw,kw. OR Eritrea.tw,kw. OR Ethiopia.tw,kw. OR Kenya.tw,kw. OR Madagascar.tw,kw. OR Mozambique.tw,kw. OR Malawi.tw,kw. OR Mauritius.tw,kw. OR Rwanda.tw,kw. OR Seychelles.tw,kw. OR Somalia.tw,kw. OR South Sudan.tw,kw. OR Tanzania.tw,kw. OR Uganda.tw,kw. OR Zambia.tw,kw. OR Zimbabwe.tw,kw. OR Benin.tw,kw. OR Burkina Faso.tw,kw. OR Cape Verde.tw,kw. OR Cabo Verde.tw,kw. OR Gambia.tw,kw. OR Ghana.tw,kw. OR Guinea.tw,kw. OR Guinea Bissau.tw,kw. OR Ivory Coast.tw,kw. OR Cote divoire.tw.kw. OR Liberia.tw,kw. OR Mali.tw,kw. OR Mauritania.tw,kw. OR Niger.tw,kw. OR Nigeria.tw,kw. OR Senegal.tw,kw. OR Sierra Leone.tw,kw. OR Togo.tw,kw. OR Angola.tw,kw. OR Cameroon.tw,kw. OR Central African Republic.tw,kw. OR Chad.tw,kw. OR Congo.tw,kw. OR DR Congo.tw,kw. OR Democratic Republic of the Congo.tw,kw. OR Republic of the Congo.tw,kw. OR Equatorial Guinea.tw,kw. OR Gabon.tw,kw. OR (Sao Tome and Principe).tw,kw. OR (Sao Tome & Principe).tw.kw. OR Botswana.tw,kw. OR Eswatini.tw,kw. OR Lesotho.tw,kw. OR Namibia.tw,kw. OR South Africa.tw,kw. | exp Africa/ or exp Africa, Eastern/ or exp Africa, Western/ or exp Africa, Southern/ or exp "Africa South of the Sahara"/ or exp Africa, Northern/ or exp Africa, Central/ OR Africa.tw,kw. OR sub?Sahara Africa.tw,kw. OR Algeria.tw,kw. OR Egypt.tw,kw. OR Libya.tw,kw. OR Morocco.tw,kw. OR Sudan.tw,kw. OR Tunisia.tw,kw. OR Burundi.tw,kw. OR Comoros.tw,kw. OR Djibouti.tw,kw. OR Eritrea.tw,kw. OR Ethiopia.tw,kw. OR Kenya.tw,kw. OR Madagascar.tw,kw. OR Mozambique.tw,kw. OR Malawi.tw,kw. OR Mauritius.tw,kw. OR Rwanda.tw,kw. OR Seychelles.tw,kw. OR Somalia.tw,kw. OR South Sudan.tw,kw. OR Tanzania.tw,kw. OR Uganda.tw,kw. OR Zambia.tw,kw. OR Zimbabwe.tw,kw. OR Benin.tw,kw. OR Burkina Faso.tw,kw. OR Cape Verde.tw,kw. OR Cabo Verde.tw,kw. OR Gambia.tw,kw. OR Ghana.tw,kw. OR Guinea.tw,kw. OR Guinea Bissau.tw,kw. OR Ivory Coast.tw,kw. OR Cote divoire.tw.kw. OR Liberia.tw,kw. OR Mali.tw,kw. OR Mauritania.tw,kw. OR Niger.tw,kw. OR Nigeria.tw,kw. OR Senegal.tw,kw. OR Sierra Leone.tw,kw. OR Togo.tw,kw. OR Angola.tw,kw. OR Cameroon.tw,kw. OR Central African Republic.tw,kw. OR Chad.tw,kw. OR Congo.tw,kw. OR DR Congo.tw,kw. OR Democratic Republic of the Congo.tw,kw. OR Republic of the Congo.tw,kw. OR Equatorial Guinea.tw,kw. OR Gabon.tw,kw. OR (Sao Tome and Principe).tw,kw. OR (Sao Tome & Principe).tw.kw. OR Botswana.tw,kw. OR Eswatini.tw,kw. OR Lesotho.tw,kw. OR Namibia.tw,kw. OR South Africa.tw,kw. | Africa (Topic) OR "sub?Sahara Africa" (Topic) OR "sub Sahara Africa" (Topic) OR Algeria (Topic) OR Egypt (Topic) OR Libya (Topic) OR Morocco (Topic) OR Sudan (Topic) OR Tunisia (Topic) OR Burundi (Topic) OR Comoros (Topic) OR Djibouti (Topic) OR Eritrea (Topic) OR Ethiopia (Topic) OR Kenya (Topic) OR Madagascar (Topic) OR Mozambique (Topic) OR Malawi (Topic) OR Mauritius (Topic) OR Rwanda (Topic) OR Seychelles (Topic) OR Somalia (Topic) OR "South Sudan" (Topic) OR Tanzania (Topic) OR Uganda (Topic) OR Zambia (Topic) OR Zimbabwe (Topic) OR Benin (Topic) OR "Burkina Faso" (Topic) OR "Cape Verde" (Topic) OR "Cabo Verde" (Topic) OR Gambia (Topic) OR Ghana (Topic) OR Guinea (Topic) OR "Guinea Bissau" (Topic) OR "Ivory Coast" (Topic) OR "Cote divoire" (Topic) OR Liberia (Topic) OR Mali (Topic) OR Mauritania (Topic) OR Niger (Topic) OR Nigeria (Topic) OR Senegal (Topic) OR "Sierra Leone" (Topic) OR Togo (Topic) OR Angola (Topic) OR Cameroon (Topic) OR "Central African Republic" (Topic) OR Chad (Topic) OR Congo (Topic) OR "DR Congo" (Topic) OR "Democratic Republic of the Congo" (Topic) OR "Republic of the Congo" (Topic) OR "Equatorial Guinea" (Topic) OR Gabon (Topic) OR "Sao Tome and Principe" (Topic) OR "Sao Tome & Principe" (Topic) OR Botswana (Topic) OR Eswatini (Topic) OR Lesotho (Topic) OR Namibia (Topic) OR "South Africa" (Topic) | Africa.tw. OR sub?Sahara Africa.tw. OR Algeria.tw. OR Egypt.tw. OR Libya.tw. OR Morocco.tw. OR Sudan.tw. OR Tunisia.tw. OR Burundi.tw. OR Comoros.tw. OR Djibouti.tw. OR Eritrea.tw. OR Ethiopia.tw. OR Kenya.tw. OR Madagascar.tw. OR Mozambique.tw. OR  Malawi.tw. OR Mauritius.tw. OR Rwanda.tw. OR Seychelles.tw. OR Somalia.tw. OR South Sudan.tw. OR Tanzania.tw. OR Uganda.tw. OR Zambia.tw. OR Zimbabwe.tw. OR Benin.tw. OR Burkina Faso.tw. OR Cape Verde.tw. OR Cabo Verde.tw. OR Gambia.tw. OR Ghana.tw. OR Guinea.tw. OR Guinea Bissau.tw. OR Cote divoire.tw. OR Ivory Coast.tw. OR Liberia.tw. OR Mali.tw. OR Mauritania.tw. OR Niger.tw. OR Nigeria.tw. OR Senegal.tw. OR Sierra Leone.tw. OR Togo.tw. OR Angola.tw. OR Cameroon.tw. OR Central African Republic.tw. OR Chad.tw. OR Congo.tw. OR DR Congo.tw. OR Democratic Republic of the Congo.tw. OR Republic of the Congo.tw. OR Equatorial Guinea.tw. OR Gabon.tw. OR (Sao Tome and Principe).tw. OR Botswana.tw. OR Eswatini.tw. OR Lesotho.tw. OR Namibia.tw. OR South Africa.tw. |  |
|  | (#1 OR #2) AND #3 AND #4 | (#1 OR #2) AND #3 AND #4 | (#1 OR #2) AND #3 AND #4 | (#1 OR #2) AND #3 AND #4 |  |
|  | limit #5 to humans | limit #5 to humans | #5 AND Review Article (Document Types) (Used to map published SR and MA) | limit #5 to humans |  |
|  | limit #6 to "review articles" | limit #6 to "systematic review" | #5 NOT #6 (Final Search) | limit #6 to "systematic review" |  |
|  | limit #6 to "systematic review" | limit #6 to "meta-analysis" |  | limit #6 to "meta-analysis" |  |
|  | limit #6 to "meta-analysis" | #7 OR #8 |  | #7 OR #8 |  |
|  | #7 OR #8 OR #9 | #6 Not #9 (Final search) |  | #6 NOT #9 (Final search) |  |
|  | #6 Not #10 (Final search) |  |  |  |  |

exp stands for explodes (used in the MeSH or EMTREE vocabulary to capture all narrower terms associated with the broader concept in Ovid Medline and Embase); tw (used to find text or word in the title and abstract field); kw (used to find texts or word in the keyword field); topic(used to find text or word in the title, abstract, and keywords field in Web of Science); *(use to include various word endings and spellings); ?(used to matches for any single alphabet in a specific position); “phrase” (used to search phrase or to find results with the exact phrase); AND (used to find results that contain both or all); OR(used to find results that contain either); and NOT(used to find results that contain the first but not the second).

- 1. **Eligibility Criteria**

**Supplementary Table S2:** Eligibility criteria

| **Inclusion** | **Exclusion** |
| --- | --- |
| - Studies must include at list one of the following HCV epidemiological reports in Sub-Saharan Africa.   - Prevalence   - Risk factors - Must use primary data/survey record review. - We take the most recent full report or abstract when we encounter duplicate reports from the same dataset | - Studies that do not contain any of the following reports.   - HCV prevalence   - HCV risk factors - Articles used secondary data or any reviews or meta-analyses. - Duplicate reports from the same dataset |
| - We included general populations:   - The general population (both sexes and all age groups),   - All community-based surveys or   - A representative sample of the entire population or   - A portion of the general population like a specific age/sex group or   - Household surveys or   - Other populations (including students) without any specific/known HCV risk referencing characteristics. | - - High-risk or Key populations (e.g., people who inject drugs, sex workers, men who have sex with men, transgender persons, etc.)   - People with possible increased risks (health care workers, refugees, prisoners, etc.)   - Studies conducted from subjects in health facilities (inpatient and outpatients, blood donors, pregnant women in antenatal care, etc.)   - Studies conducted from subjects in comorbid/chronic illnesses (haemodialysis, sickle cell diseases, haemophilia, HIV, diabetics, liver diseases, tuberculosis, etc.)   - People with different demographic characteristic with the general population such as factory workers, migrants, etc. |
| - We included:   - Observational (cohort, cross-sectional, and case-control) studies | - Study in non-human subjects. - Studies on diagnostic and/or laboratory method performance. |
| - There is no sample size restriction. - There is no publication date restriction. - There is no publication language restriction. |  |
| - Articles reporting data from one or more of Sub-Saharan Africa country(s) and/or any of their regions/districts. | - Studies conducted in Egypt, Morocco, Libya, Tunisia, and Algeria |
| - We manually searched the reference list of relevant published reviews, systematic reviews, or meta-analyses in the topic. |  |

- 1. **Critical appraisal**

We used the Joanna Briggs Institute critical appraisal tool for prevalence studies to assess the quality of included articles or abstracts or short reports (1). This tool has nine questions to evaluate the internal and external validity of the study with a possible answer of “Yes”, “No”, “Unclear”, or “Not applicable” for each question based on the information reported in the article or the data provided during the email communication within the authors of the study. The first four questions are important to assess external validity on sampling frame appropriateness, study subject selection method, sample size adequacy, and description of study subjects and setting. The remaining five questions are used to assess the internal validity of the study which enables us to assess sufficient coverage of data analysis for all subgroups, the use of proper statistical analysis, the use of valid methods to identify HCV, the measure of HCV in a standard/reliable way, and the adequacy of the response rate. Like the published articles we also critical appraised abstracts or short reports based on the information they have. However, they had limited information to say yes or no, and in most of the questions we classify them as unclear (see **Supplementary Table S3)**. Finally, we summed the number of yeses for each study scored out of these nine questions and classified those articles scored eight or nine yes as high quality.

**Supplementary Table S3:** Joanna Briggs Institute critical appraisal criteria

| **Questions** | **Yes** | **No** | **Unclear** |
| --- | --- | --- | --- |
| 1. Was the sample frame appropriate to address the target population? | - If the included population are almost all members of the target population. - If the sampling frame is representative of the target population in terms of broader population characteristics and geographical area. | - If the sampling frame is not representative of the target population or unrepresentative group of the general population are considered as target population | If we didn’t get, clear information on the sampling frame or the target population to say yes or no. |
| 2. Were study participants sampled in an appropriate way? | When sampling the study population from the sampling frame is probabilistic/representative.   - Random sampling - Census - Cluster sampling/random sample of villages. | - When sampling the study population from the sampling frame is nonprobability such as voluntary survey, convenience samples, and quota survey. - When unrepresentative samples of the target population had been taken and the results was inferred to the target population | If they didn’t report how they sampled the study population, or their description is not enough to say yes or no. |
| 3. Was the sample size adequate? | - When it is large national surveys. - When the sample size is above 400 and it is Censes. - When they used appropriate sample size calculation formula, and the sample size is above 400. | - When sample size is below 400 - When they didn’t calculate the sample size using appropriate statistical formula or censes the whole sampling frame. - When there is no sample size calculation, and it is not a large national survey. | When the sample size is above 400 and unable to figure out the appropriateness of sample size estimation methods. |
| 4. Were the study subjects and the setting described in detail? | If they clearly describe the study area related to urban/rural, districts, Zones, province/state, and country name; and populations size and characteristics. | They say few things about the study population or geographical characteristics and if we can’t sure about the comparability with the general population interest. | If they didn’t say anything about the geographic area or we can’t get adequate information to say yes or no. |
| 5. Was the data analysis conducted with sufficient coverage of the identified sample? | If they clearly reported how they estimated seroprevalence and viraemic prevalence.  If the reported prevalence aligns with our calculation of (Numerator*100% / Denominator) | - If they didn’t correctly estimate the prevalence. | In their report if they miss one of the following   - Prevalence, - Numerator, or - Denominator |
| 6. Were valid methods used for the identification of the condition? | - If they used either ELISA or RDT to test HCV serology and HCV RNA for virological testing - And if the sample is either whole blood or plasma or serum or DBS | ____ | If they didn’t report the type of HCV tests they used, or the blood sample they tested. |
| 7. Was the condition measured in a standard, reliable way for all participants? | If they clearly follow and reported Standard Operating Procedures (SOPs).   - How blood sample was collected - How the testing is conducted - How quality (result validity) was controlled - When they say the result are positive or negative or indeterminant - How they follow the test kit manufacturers instruction - Who performed the test? - Was the measurement conducted in the same way for all participants | If they didn’t follow any SOPs or the manufacturer instruction to test. | If they didn’t clearly state   - How blood samples are collected and stored - How the test was performed and - How the result was interpreted |
| 8. Was there appropriate statistical analysis? | If they clearly reported both the numerator and denominator to calculate HCV seroprevalence or viraemia prevalence. | If they report HCV positive percentage but not both the denominator or numerator. | If they report the percentage of HCV positive with either the denominator or numerator. |
| 9. Was the response rate adequate, and if not, was the low response rate managed appropriately? | - If the response rate is >85% - If the response rate is below 85% and managed appropriately.   - Done sensitivity analysis and the result didn’t affect the non-responders.   - Adequately justify that the non-response appears to be unrelated to the outcome.   - Socio-demographic and other characteristics of responded are comparable with the non-responders.   - Consider adequate number of nonresponse rate during the sample size calculation.   - Substituting next household/samples for none-responders in the case of household survey. | If nonresponse rate from the selected sample is greater than 15% and not properly managed. | If we didn’t get enough data to calculate the response rate or how they managed it. |

- 1. **Age classification**

It was not possible to get a common age grouping across the included studies. The common age group reported across the studies was 20 and below, 21 to 64, and above 64 years old. There was also an age grouping that crossed the above age cutting value. For those having the lower and upper age range, we calculated the midpoint and categorized according to which this midpoint lays. We grouped those studies that reported the lower age is from 15 to 49 but lacks the maximum age into the age group 21 to 64 thinking most of the population in these categories might be the adult population. For those studies saying age above 49, we grouped in the age group >65 by considering most of the population would be older (see **Supplementary Table S4**).

**Supplementary Table S4**: Age categorization in years

| Ages grouped under <20 | Ages grouped under 21 to 64 | Ages grouped under >65 |
| --- | --- | --- |
| < 20 | 21 to 64 | >65 |
| < 25, 0 to 35, 1 to 22, 1 to 34, 1 to 39, 2 to 24, 12 to 24, 13 to 24, 15 to 24, 15 to 25, 16 to 24, and 18 to 22 | >15, >16, >20, >24, ≥26, >29, >30, >34, >35, >40, >44, >45, 2 to 50, 5 to 40, 12 to 80, 13 to 78, 15 to 29, 15 to 30, 15 to 34, 15 to 70, 16 to 25, 16 to 40, 16 to 100, 18 to 24, 18 to 25, 18 to 27, 18 to 29, 18 to 35, 18 to 45, 19 to 40, 20 to 24, 20 to 29, 24 to 71, 30 to 69, 41 to 97, 46 to 65, 56 to 77, 58 to 67, and 58 to 68 | >49, >50, >51, >54, >55, >57, >60, 51 to 100, 55 to 90, 60 to 75, 61 to 70, and 60 to 90 |

- 1. **Classifications of SSA countries into subregions**

We grouped the SSA countries into four sub-regions based on the WHO geography classification and the actual geographical location of each country: Africa-Eastern, Africa-Western, Africa-Southern, and Africa-Central (see **Supplementary Table S5).**

**Supplementary Table S5**: Classifications of SSA countries into subregions

| **SSA subregions** | **Name of countries** |
| --- | --- |
| Africa-Western | Nigeria, Burkina Faso, Guinea, Benin, Senegal, Sierra Leone, Mali, Ghana, Guinea Bissau, Gambia, Cabo Verde, Cote d’Ivoire, Liberia, Mauritania, Niger, and Togo |
| Africa-Central | Cameroon, Democratic Republic of the Congo (DR Congo), Equatorial Guinea, Central Africa Republic, Chad, Republic of the Congo, Gabon, and Sao Tome and Principe |
| Africa-Eastern | Ethiopia, Tanzania, Kenya, Rwanda, Uganda, Madagascar, Reunion, Burundi, Sudan, Eritrea, Mayotte, Seychelles, Somalia, South Sudan, Comoros, Djibouti, and Mauritius |
| Africa-Southern | Malawi, South Africa, Zambia, Zimbabwe, Eswatini (formerly named Swaziland), Lesotho, Angola, Botswana, Namibia, and Mozambique |

- 1. **Weighted overall and subregional analysis**

Following having the best estimate for each country (see **Supplementary Table S6**), a weighted overall or subregional HCV seroprevalence was estimated using a weighted mean by accounting for the country-specific HCV seroprevalence estimates and the 2021 United Nations population estimates of each country population size.

**Supplementary Table S6**: Criteria used to select best estimates for each country in SSA

| Choice | Best data for HCV seroprevalence estimate of each country | |
| --- | --- | --- |
| 1^st^ | National estimate from a household survey | We used the study with largest representative sample size unless there is a smaller study that is either i) much more recent, or ii) has a much more comprehensive age, sex, and geography distribution |
| 2^nd^ | Multi-site study |  |
| 3^rd^ | Multiple single-site studies for a given country | We used estimates from the fixed-effects meta-analysis using high-quality studies from the JBI critical assessment |
| 4^th^ | One single-site study for a given country | We used it as it is |
| 5^th^ | For 18 countries without data and Eritrea with zero prevalence in the included studies. | For 13 countries including Eritrea, we imputed seroprevalence reports from the World Health Organization’s (WHO) African region hepatitis scorecard for each country (2). Six countries had no seroprevalence data in the WHO African region report, and we imputed the best available data from their neighbouring or nearest country. These six countries are Chad, Djibouti, Liberia, Reunion, Mauritania, and Eswatini; we imputed data from Central Africa Republic, Ethiopia, Guinea, Madagascar, Mali, and South Africa, respectively. |

1. **Results**
   1. **Pooled HCV seroprevalence from the random-effects meta-analysis**

The overall pooled HCV seroprevalence using all the included studies in SSA was 4.17% (95% CI: 3.71-4.66). The pooled estimates in the four subregions of SSA were: 7.61% (95% CI: 5.33-10.26) in Africa-Central, 3.68% (95% CI: 3.11-4.30) in Africa-Eastern, 3.37% (95% CI: 2.38-4.52) in Africa-Western, and 1.63% (95% CI: 0.41-3.54) in Africa-Southern. The estimated pooled HCV seroprevalence in the three countries contributing the greatest number of datasets were Ethiopia (1.56%, 95% CI: 0.87-2.43), Nigeria (3.73%, 95% CI: 2.11-5.75), and Tanzania (6.75%, 95% CI: 2.08-13.72). There was substantial heterogeneity (I^2^>90%) throughout the analysis **(see Supplementary Table S7)**.

**Supplementary Table S7**: Pooled HCV seroprevalence in Sub-Saharan Africa, its subregions, and countries using all included studies from the random-effects meta-analysis.

| Subgroup | Number of datasets | Total participants with valid result | Total HCV seropositive participants | Pooled HCV seroprevalence (95% CI) | Heterogeneity (I^2^) | Q-statistics (p. value) |
| --- | --- | --- | --- | --- | --- | --- |
| **SSA** | 136 | 1679755 | 94740 | 4.17 (3.71-4.66) | 99.30 | <0.01 |
| **Africa-Eastern** | 49 | 1525058 | 88846 | 3.68 (3.11-4.30) | 99.50 | <0.01 |
| Tanzania | 9 | 5248 | 276 | 6.75 (2.08-13.72) | 98.48 | <0.01 |
| Kenya | 5 | 3231 | 95 | 3.03 (0.85-6.37) | 94.08 | <0.01 |
| Ethiopia | 11 | 14854 | 307 | 1.56 (0.87-2.43) | 91.37 | <0.01 |
| Madagascar | 4 | 3800 | 58 | 1.57 (0.72-2.69) | 74.28 | <0.01 |
| Eritrea | 1 | 45 | 0 | 0.00 (0.00-7.87) | -- | -- |
| South Sudan | 1 | 158 | 5 | 3.16 (1.36-7.19) | -- | -- |
| Somalia | 1 | 672 | 9 | 1.34 (0.71-2.53) | -- | -- |
| Rwanda | 6 | 1469883 | 86949 | 10.05 (7.92-12.40) | 99.93 | <0.01 |
| Mayotte | 1 | 2917 | 6 | 0.21 (0.09-0.45) | -- | <0.01 |
| Uganda | 5 | 16976 | 656 | 5.36 (3.91-7.02) | 91.74 | <0.01 |
| Sudan | 2 | 609 | 12 | 1.94 (0.95-3.24) | -- | -- |
| Burundi | 2 | 5659 | 468 | 8.05 (7.34-8.78) | -- | -- |
| Seychelles | 1 | 1006 | 5 | 0.50 (0.21-1.16) | -- | -- |
| **Africa-Central** | 31 | 64158 | 2877 | 7.61 (5.33-10.26) | 99.13 | <0.01 |
| Central Africa Republic | 3 | 5756 | 130 | 3.04 (0.00-10.89) | -- | -- |
| Cameroon | 17 | 46756 | 1798 | 9.19 (5.82-13.21) | 99.23 | <0.01 |
| DR Congo | 2 | 2148 | 229 | 6.98 (5.94-8.10) | -- | -- |
| Republic of the Congo | 1 | 887 | 50 | 5.64 (4.30-7.35) | -- | -- |
| Gabon | 5 | 6017 | 628 | 10.90 (6.47-16.29) | 95.68 | <0.01 |
| Equatorial Guinea | 3 | 2594 | 42 | 1.52 (1.07-2.05) | -- | -- |
| **Africa-Western** | 50 | 86904 | 2960 | 3.37 (2.38-4.52) | 98.50 | <0.01 |
| Guinea Bissau | 2 | 4314 | 89 | 1.61 (1.25-2.01) | -- | -- |
| Guinea | 4 | 1818 | 30 | 4.14 (0.77-9.53) | 90.89 | <0.01 |
| Sierra Leone | 1 | 66 | 1 | 1.52 (0.27-8.10) | -- | -- |
| Nigeria | 29 | 46730 | 1964 | 3.73 (2.11-5.75) | 98.98 | <0.01 |
| Mali | 2 | 678 | 16 | 2.26 (1.24-3.56) | -- | -- |
| Burkina Faso | 5 | 17449 | 625 | 2.75 (1.62-4.17) | 88.16 | <0.01 |
| Gambia | 2 | 2928 | 22 | 0.61 (0.34-0.94) | -- | -- |
| Ghana | 2 | 941 | 66 | 6.69 (5.16-8.40) | -- | -- |
| Benin | 2 | 11844 | 143 | 1.20 (1.01-1.40) | -- | -- |
| Senegal | 1 | 136 | 4 | 2.94 (1.15-7.32) | -- | -- |
| **Africa-Southern** | 6 | 3635 | 57 | 1.63 (0.41-3.54) | 91.38 | <0.01 |
| South Africa | 1 | 617 | 7 | 1.13 (0.55-2.32) | -- | -- |
| Zimbabwe | 1 | 269 | 1 | 0.37 (0.07-2.08) | -- | -- |
| Malawi | 3 | 2356 | 43 | 2.51 (0.03-8.16) | -- | -- |
| Zambia | 1 | 393 | 6 | 1.53 (0.70-3.29) | -- | -- |

SSA: Sub-Saharan Africa; CI: Confidence Interval; I^2^: Heterogeneity; HCV: Hepatitis C Viruses.

- 1. **Pooled HCV seroprevalence using studies that used Enzyme Linked Immunosorbent Assay/Enzyme Immune Assay testing methods in the random-effect model.**

From those 99 studies used Enzyme Linked Immunosorbent Assay/Enzyme Immune Assay testing methods, the overall pooled HCV seroprevalence was 4.67% (95% CI: 4.16-5.21) and it was 9.55% (95% CI: 6.00-13.81), 4.03% (95% CI: 2.74-5.53), 3.47% (95% CI: 2.96-4.02), 1.63% (95% CI: 0.41-3.54) in Africa-Central, Africa-Western, Africa-Eastern, and Africa-Southern, respectively. Heterogeneity was >90% for all estimates.

- 1. **Pooled HCV seroprevalence using high-quality studies in the random-effect model.**

Across high-quality studies, the pooled HCV seroprevalence was 3.41% (95% CI: 2.87-3.99) for the whole SSA, and 2.65% (95% CI: 2.06-3.31), 2.72% (95% CI: 1.55-4.20), and 6.27% (95% CI: 3.28-10.13) in Africa-Eastern, Africa-Western, and Africa-Central, respectively. There were no high-quality studies for Africa-Southern. The highest seroprevalence by country was in Gabon (11.63%, 95% CI: 7.06-17.15), followed by Cameroon (7.17%, 95% CI: 2.41-14.16), and Rwanda (5.82%, 95% CI: 4.07-7.84) (see **Supplementary Table S8**).

**Supplementary Table S8**: Pooled seroprevalence of HCV in Sub-Saharan Africa using high-quality studies from the random-effects meta-analysis.

| Subgroup | Number of datasets | Total participants with valid result | Total HCV seropositive participants | Pooled seroprevalence (95% CI) | Heterogeneity (I^2^) | Q-statistics (p-value) |
| --- | --- | --- | --- | --- | --- | --- |
| **By geography** | | | | | | |
| **SSA** | 53 | 1594515 | 90400 | 3.41 (2.87-3.99) | 99.59 | <0.01 |
| **Africa-Eastern** | 24 | 1510727 | 87626 | 2.65 (2.06-3.31) | 99.70 | <0.01 |
| Tanzania | 2 | 2419 | 96 | 3.78 (3.05-4.59) | -- | -- |
| Kenya | 2 | 2399 | 60 | 2.12 (1.58-2.74) | -- | -- |
| Ethiopia | 7 | 9012 | 105 | 1.24 (0.92-1.61) | 37.50 | 0.14 |
| Madagascar | 2 | 3071 | 36 | 1.17 (0.81-1.58) | -- | -- |
| Rwanda | 4 | 1467786 | 86248 | 5.82 (4.07-7.84) | 99.95 | <0.01 |
| Mayotte | 1 | 2917 | 6 | 0.21 (0.09-0.45) | -- | -- |
| Uganda | 3 | 16349 | 609 | 4.61(3.31-6.11) | -- | -- |
| Sudan | 1 | 199 | 3 | 1.51 (0.51-4.34) | -- | -- |
| Burundi | 1 | 5569 | 458 | 8.22 (7.53-8.97) | -- | -- |
| Seychelles | 1 | 1006 | 5 | 0.50 (0.21-1.16) | -- | -- |
| **Africa-Central** | 14 | 31846 | 1377 | 6.27 (3.28-10.13) | 99.24 | <0.01 |
| Central Africa Republic | 2 | 4851 | 35 | 0.68 (0.46-0.94) | -- | -- |
| Cameroon | 8 | 19453 | 715 | 7.17 (2.41-14.16) | 99.26 | <0.01 |
| Gabon | 3 | 5500 | 592 | 11.63(7.06-17.15) | -- | -- |
| Equatorial Guinea | 1 | 2042 | 35 | 1.71(1.23-2.37) | -- | -- |
| **Africa-Western** | 15 | 51942 | 1397 | 2.72 (1.55-4.20) | 98.74 | <0.01 |
| Guinea Bissau | 2 | 4314 | 89 | 1.61 (1.25-2.01) | -- | -- |
| Guinea | 1 | 1253 | 11 | 0.88 (0.49-1.57) | -- | -- |
| Nigeria | 8 | 25602 | 659 | 3.58 (1.45-6.60) | 99.12 | <0.01 |
| Burkina Faso | 2 | 15366 | 583 | 3.75 (3.45-4.06) | -- | -- |
| Gambia | 1 | 2598 | 13 | 0.50 (0.29-0.85) | -- | -- |
| Benin | 1 | 2809 | 42 | 1.50 (1.11-2.01) | -- | -- |
| **Africa-Southern** | 0 | -- | -- | -- | -- | -- |

SSA: Sub-Saharan Africa; CI: Confidence Interval; I^2^: Heterogeneity; HCV: Hepatitis C Virus

- 1. **HCV seroprevalence by calendar period from the random-effects model**

From the overall included studies, there were 33 studies which didn’t report the study year. Of those, we grouped nine studies published before 2000 into the calendar period 1984-2000, two studies published in 2015 and 11 studies published from 2003-2014 into the calendar period 2001-2014, two studies published in 2016 and nine studies published from 2018 and onwards are grouped under the calendar period 2015-2023. We grouped those studies without study years assuming that the data collection period for most of the studies is one year prior to their publication year.

**Supplementary Table S9:** Seroprevalence of HCV among the general population of Sub-Saharan Africa by the calendar period from the random-effects meta-analysis.

| **Calendar Period** | **1984 to 2000** | | **2001 to 2014** | | **2015 to 2023** | |
| --- | --- | --- | --- | --- | --- | --- |
|  | **n/ N** | **P (95% UI), k** | **n/ N** | **P (95% UI), k** | **n/ N** | **P (95% UI), k** |
| **For overall studies** | | | | | | |
| SSA | 1377 / 26969 | 5.74 (3.87-7.92)^a^, 36 | 5670/ 138507 | 4.35 (3.31-5.51)^a^, 61 | 87693/ 1514279 | 3.03 (2.42-3.70)^a^, 39 |
| Africa-Eastern | 461 / 15487 | 4.00 (2.03-6.54)^a^, 17 | 1368 / 33156 | 3.32 (2.19-4.67)^a^, 16 | 87017/ 1476415 | 4.33 (3.34-5.43)^a^, 16 |
| Africa-Western | 84 / 2175 | 3.46 (1.59-5.94)^c^, 6 | 2282 / 50705 | 4.13 (2.47-6.17)^a^, 27 | 594/ 34024 | 2.14 (1.30-3.15)^a^, 17 |
| Africa-Central | 825 / 8690 | 10.60 (6.51-15.52)^a^, 12 | 1983/ 53289 | 7.08 (4.02-10.91)^a^, 14 | 69 / 2179 | 3.27 (1.06-6.55)^b^, 5 |
| Africa-Southern | 7 / 617 | 1.13 (0.55-2.32)^d^, 1 | 37 / 1357 | 2.11 (0.05-6.48)^b^, 4 | 13 /1661 | 0.78 (0.46-1.33)^d^, 1 |
| **For high quality studies** | | | | | | |
| Sub-Saharan Africa | 693 / 14356 | 4.66 (1.91-8.51)^a^, 12 | 3003/ 86312 | 3.57 (2.39-4.98)^a^, 24 | 86704/ 1493847 | 2.58 (1.90-3.37)^a^, 17 |
| **Four countries with better data across the calendar periods** | | | | | | |
| Cameroon | 683/ 4866 | 13.72 (13.7-21.07)^e^, 8 | 1053/ 40263 | 6.32 (2.60-11.49)^a^, 6 | 62/1627 | 5.31 (1.17-12.01)^d^, 3 |
| Burkina Faso | 20/ 638 | 3.13(2.04-4.79)^d^, 1 | 575/ 15881 | 3.55 (3.27-3.85)^d^, 2 | 30/930 | 3.20 (2.15-4.45)^d^, 2 |
| Ethiopia | 230/ 9759 | 1.58(0.16-4.29)^d^,3 | 23/1707 | 1.35 (0.90-2.01)^d^, 1 | 54/ 3388 | 1.56 (1.15-2.01)^f^, 7 |
| Nigeria | No data | No data | 1570/ 28395 | 4.80 (2.24-8.22)^a^, 19 | 394/ 18335 | 2.10 (0.85-3.85)^a^, 10 |
| Tanzania | 162/ 1675 | 13.15 (1.26-34.25)^a^,5 | 114/ 3573 | 1.81 (0.20-4.84)^a^,4 | No data | No data |

SSA: Sub-Saharan Africa; n: number of HCV seropositive; N: number of tested for HCV serology; P= pooled seroprevalence; CI: Confidence Interval; k: number of pooled studies; ^a^ I^2^ >95%; ^b^ I^2^ 90-95%; ^c^ I^2^ 80-90%; ^d^I^2^ not applicable; ^e^I^2^=58.74; ^f^I^2^=00.00. All Q statistic P-values were <0.01. We didn’t pool HCV seroprevalence for the Africa-Southern region in the years 1984 to 2000 and 2015 to 2023 because there was only one study.


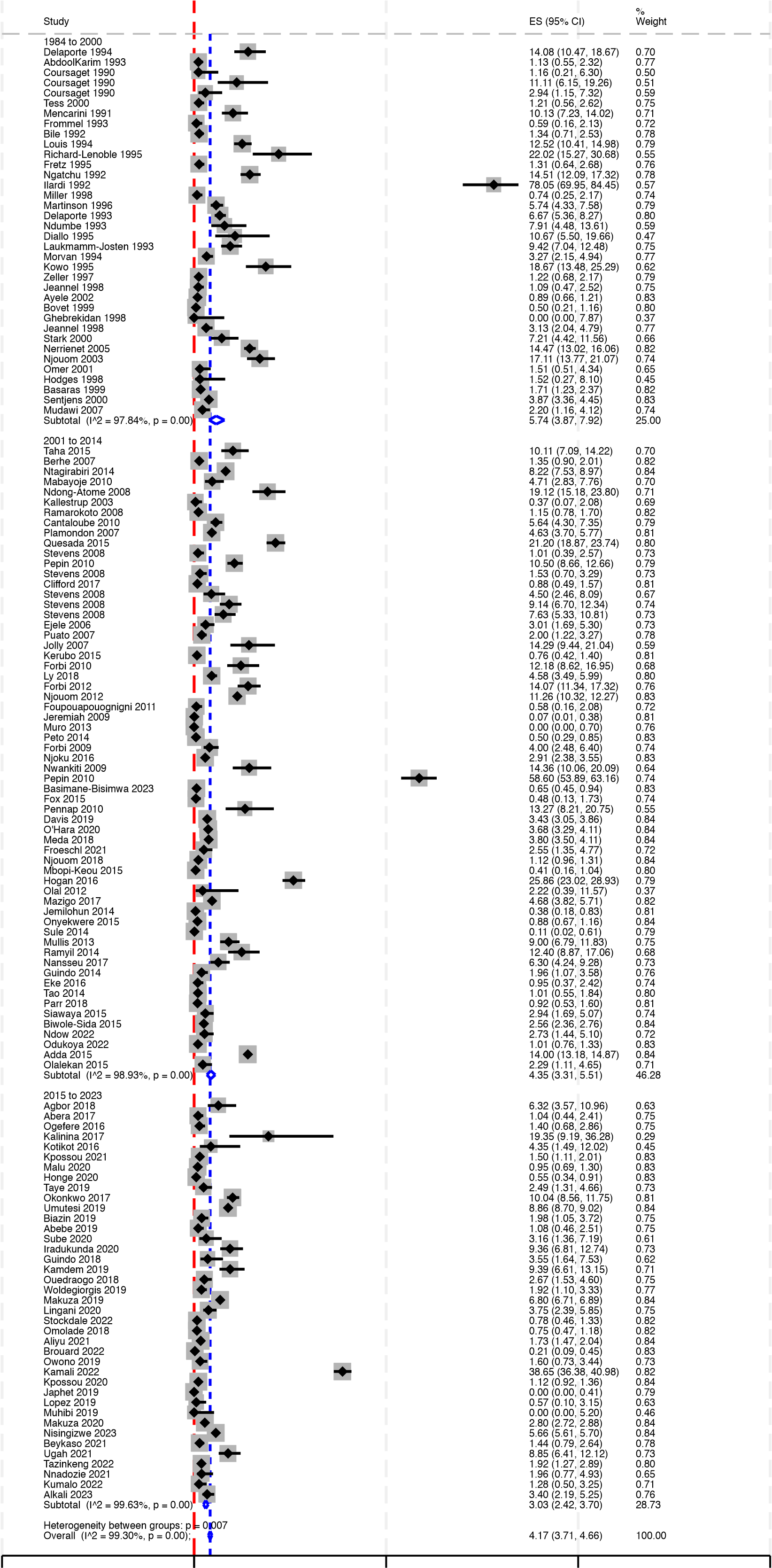


-50 0 50 100 150

**Supplementary Figure 1**: Forest plot for HCV seroprevalence in Sub-Saharan Africa stratified by calendar period.

- 1. **HCV viraemia prevalence in the random-effects model**

**Supplementary Table S10**: Viraemic prevalence of HCV in Sub-Saharan Africa overall, by Subregions, and countries from the random-effects meta-analysis.

|  | **HCV viraemic prevalence among the general population** | | **HCV viraemic prevalence among HCV seropositive** | |
| --- | --- | --- | --- | --- |
|  | **n/N** | **P (95% CI), k** | **n/N** | **P (95% CI), k** |
| **For overall studies by regions and study years** | | | | |
| **SSA** | **31807/1193586** | **2.64 (2.11-3.23)^a^, 32** | **31610 /54314** | **54.77 (47.80-61.66)^a^, 30** |
| **Africa-Eastern** | **30835/1167427** | **1.56 (1.03-2.21)^a^, 13** | **30835/53133** | **41.54 (32.35-51.02)^a^, 13** |
| Tanzania | 3/1245 | 0.24 (0.02-0.62)^d^, 2 | 3/21 | 13.56 (0.92-33.51)^d^, 2 |
| Kenya | 0/1091 | 0.00 (0.00-0.35)^d^, 1 | 0/40 | 0.00 (0.00-8.76)^d^, 1 |
| Madagascar | 17/2169 | 0.78 (0.49-1.25)^d^, 1 | 17/36 | 47.22 (31.99-62.99)^d^, 1 |
| Rwanda | 30741/1143246 | 5.47 (4.08-7.04)^a^, 4 | 30741/ 52583 | 67.49 (58.19-76.14)^a^, 4 |
| Mayotte | 3/2917 | 0.10 (0.03-0.30)^d^, 1 | 3/6 | 50.00 (18.76-81.24)^d^, 1 |
| Uganda | 71/16349 | 1.47 (0.22-3.72)^d^, 3 | 71/438 | 40.90 (0.05-94.50)^a^, 3 |
| Sudan | 0/410 | 0.00 (0.00-0.93)^d^, 1 | 0/9 | 0.00 (0.00-29.91)^d^, 1 |
| **Africa-Central** | **823/18057** | **5.89 (2.93-9.78)^a^, 11** | **626/963** | **71.10 (49.89-88.81)^a^, 9** |
| Central Africa Republic | 83/1439 | 4.93 (3.87-6.12)^d^, 2 | 83/102 | 83.16 (74.48-90.55)^d^, 2 |
| Cameroon | 409/8922 | 12.31 (0.00-45.28)^d^, 3 | 227/265 | 73.77 (36.97-99.02)^d^, 3 |
| DR Congo | 15/1309 | 1.15 (0.70-1.88)^d^, 1 | -- | -- |
| Republic of the Congo | 37/887 | 3.49 (2.47-4.92)^d^, 1 | 31/50 | 62.00 (48.15-74.14)^d^, 1 |
| Gabon | 285/5500 | 6.64 (1.63-14.63)^d^, 3 | 285/546 | 68.66 (25.17-98.37)^d^, 3 |
| **Africa-Western** | **146/6441** | **2.06 (0.30-5.05)^a^, 7** | **146/205** | **59.86 (27.39-88.62)^b^, 7** |
| Guinea Bissau | 76/4314 | 1.33 (1.00-1.69)^d^, 2 | 76/84 | 91.75 (84.30-97.24)^d^, 2 |
| Guinea | 6/490 | 0.53 (0.00-1.63)^d^, 2 | 6/10 | 66.41 (31.80-94.52)^d^, 2 |
| Nigeria | 60/519 | 11.56 (9.09-14.60)^d^, 1 | 60/73 | 82.19 (71.88-89.29)^d^, 1 |
| Burkina Faso | 4/1118 | 0.35 (0.06-0.81)^d^, 2 | 4/38 | 10.12 (1.81-22.57)^d^, 2 |
| **Africa-Southern** | **3/1661** | **0.18 (0.06-0.53)^d^, 1** | **3/13** | **23.08 (8.18-50.26)^d^, 1** |
| Malawi | 3/1661 | 0.18 (0.06-0.53)^d^, 1 | 3/13 | 23.08 (8.18-50.26)^d^, 1 |
| **By calendar periods** | | | | |
| 2015 to 2023 | 30943/1158787 | 2.01(1.08-3.18)^a^, 8 | 30761/52640 | 59.04 (50.26-68.57)^a^, 9 |
| 2001 to 2014 | 789/30715 | 4.59 (2.17-7.82)^a^, 16 | 774/1,528 | 55.62 (30.41-79.49)^a^, 14 |
| 1984 to 2000 | 75/4084 | 1.26 (0.14-3.30)^b^, 7 | 75/146 | 42.37 (17.08-69.63)^c^, 7 |
| **For high quality studies by subregions** | | | | |
| SSA | 30800/1176127 | 1.67 (1.24-2.17)^a^, 19 | 30800/53299 | 51.86 (43.82-59.86)^a^, 19 |
| Africa-Eastern | 30385/1164544 | 0.97 (0.57-1.47)^a^, 10 | 30385/52572 | 38.64 (22.40-56.16)^a^ , 9 |
| Africa-Western | 77/4794 | 1.13 (0.00-4.27)^d^, 3 | 77/102 | 60.15 (5.01-100.00)^d^, 3 |
| Africa-Central | 338/6789 | 4.32 (1.26-9.01)^a^, 6 | 338/625 | 63.87 (35.12-88.71)^a^, 6 |
| **For high quality studies by calendar periods** | | | | |
| 2015 to 2023 | 30308/1147635 | 1.08 (0.66-1.61)^a^, 6 | 29893/52084 | 57.11 (47.98-66.03)^a^, 6 |
| 2001 to 2014 | 424/25915 | 2.31 (0.70-4.78)^a^, 9 | 424/1102 | 47.09 (16.54-78.76)^a^, 9 |
| 1984 to 2000 | 68/2577 | 2.28 (0.10-6.85)^a^, 4 | 68/113 | 54.59 (33.79-74.69)^e^, 4 |

SSA: Sub-Saharan Africa; n: number of HCV seropositive; N: number of tested for HCV serology; P: pooled viraemic prevalence; CI: Confidence Interval; k: number of pooled studies; ^a^I^2^>95% ^b^ I^2^ 90-95% ^c^I^2^70-89% ^d^I^2^ not applicable ^e^I^2^=66.37%. All Q static P-value were <0.01, except ^d^ which is 0.03. We can’t estimate the viraemic prevalence of HCV in the Africa-Southern region because the number of included studies reporting HCV viraemia among the general population was only one.

- 1. **Responses to the nine Joanna Briggs Institute quality assessment questions**

**Supplementary Figure 2:** Number of yes, no, and unclear responses to each Joanna Briggs Institute quality assessment questions (n=137).

- 1. **Included studies characteristics.**

**Supplementary Table S11:** Main characteristics of the included studies in the meta-analysis.

| **Study ID** | **Title** | **Publication Type** | **Country by subregions** | **Study Design** | **Study population** | **Sampling method** | **Study period** | **Diagnosis Method** | **JBI Quality** |
| --- | --- | --- | --- | --- | --- | --- | --- | --- | --- |
|  |  |  | **Western** |  |  |  |  |  |  |
| Kpossou 2021 | [Seroprevalence of hepatitis C virus (HCV) antibodies and associated factors based on voluntary screening data collection in the general population in Benin in 2016] | Full Text | Benin | Cross-sectional | Mixed | None-probability | 2016 | RDT | High |
| Kpossou 2020 | [Seroprevalence of viral hepatitis B and its associated factors determined based on data from a screening campaign targeting the general population in Benin] OR  Viral hepatitis B during a general population screening campaign in Benin: seroprevalence and associated factors | Full Text | Benin | Cross-sectional | Mixed | None Probability | 2019 | Unspecified | Poor |
| Ouedraogo 2018 | Prevalence of HIV, hepatitis B and hepatitis C infection among potential participants to experimental vaccines trials in a rural area of Burkina Faso | Abstract | Burkina Faso | Cross-sectional | Adult | None Probability | 2018 | RDT | Poor |
| Lingani 2020 | The changing epidemiology of hepatitis B and C infections in Nanoro, rural Burkina Faso: a random sampling survey | Full Text | Burkina Faso | Cross-sectional | Children | Probability | 2018 | ELISA & RNA-PCR | High |
| Tao 2014 | Seroepidemiology of hepatitis B and C viruses in the general population of Burkina Faso | Full Text | Burkina Faso | Cross-sectional | Mixed | None Probability | 2014 | RDT | Poor |
| Meda 2018 | Hepatitis B and C virus seroprevalence, Burkina Faso: a cross-sectional study | Full Text | Burkina Faso | Cross-sectional | Adult | Probability | 2011 | ELISA/EIA | High |
| Ndow 2022 | Occult Hepatitis B infection is frequent and a risk factor of advanced liver disease in The Gambia, West Africa OR  Prevalence and Clinical Significance of Occult Hepatitis B Infection in The Gambia, West Africa | Full Text | Gambia | Cross-sectional | Mixed | Probability | 2014 | RDT | Poor |
| Peto 2014 | Efficacy and effectiveness of infant vaccination against chronic hepatitis B in the Gambia Hepatitis Intervention Study (1986-90) and in the nationwide immunisation program | Full Text | Gambia | Cross-sectional | Adult | Probability | 2008 | ELISA/EIA | High |
| Jolly 2007 | Association between aflatoxin exposure and health characteristics, liver function, hepatitis and malaria infections in Ghanaians | Full Text | Ghana | Cross-sectional | Mixed | None Probability | 2007 | ELISA/EIA | Moderate |
| Martinson 1996 | Seroepidemiological survey of hepatitis B and C virus infections in Ghanaian children | Full Text | Ghana | Cross-sectional | Student | Probability | 1993 | ELISA/EIA | Moderate |
| Diallo 1995 | Prevalence of exposure to aflatoxin and hepatitis B and C viruses in Guinea, West Africa | Full Text | Guinea | Cross-sectional | Adult Male | Unspecified | 1993 | ELISA/EIA | Poor |
| Clifford 2017 | Hepatitis C virus seroprevalence in the general female population of 9 countries in Europe, Asia and Africa | Full Text | Guinea | Cross-sectional | Adult Women | None Probability | 2006 | ELISA/EIA | High |
| Kalinina 2017 | THE OCCURRENCE OF THE MARKERS OF HEPATITIS C AMONG PRACTICALLY HEALTHY RESIDENTS OF THE REPUBLIC OF GUINEA: A PILOT STUDY | Full Text | Guinea | Cross-sectional | Mixed | Probability | 2016 | ELISA & RNA-PCR | Poor |
| Honge 2020 | Hepatitis B and C in the adult population of Bissau, Guinea-Bissau: a cross-sectional survey | Full Text | Guinea Bissau | Cross-sectional | Mixed | Probability | 2016 | ELISA & RNA-PCR | High |
| Plamondon 2007 | Hepatitis C virus infection in Guinea-Bissau: a sexually transmitted genotype 2 with parenteral amplification? | Full Text | Guinea Bissau | Cross-sectional | Old | None Probability | 2005 | ELISA & RNA-PCR | High |
| Hodges 1998 | Seroprevalence of hepatitis markers; HAV, HBV, HCV and HEV amongst primary school children in Freetown, Sierra Leone | Short report | Sierra Leone | Cross-sectional | Student | None Probability | 1998 | Unspecified | Poor |
| Guindo 2018 | Causes of screening failure during a transmission blocking vaccine trial around Doneguebougou, Mali | Abstract | Mali | Cross-sectional | Adult | None Probability | 2017 | Unspecified | Poor |
| Guindo 2014 | Hepatitis b, hepatitis c and HIV infection frequencies among volunteers screened for malaria vaccine clinical trials in Mali | Abstract | Mali | Cross-sectional | Adult | None Probability | 2014 | ELISA/EIA | Poor |
| Jemilohun 2014 | Prevalence of Hepatitis C virus antibody among undergraduates in Ogbomosho, South-Western Nigeria | Full Text | Nigeria | Cross-sectional | Student | Probability | 2012 | RDT | Moderate |
| Mabayoje 2010 | Prevalence of hepatitis B surface antigen, hepatitis C and human immunodeficiency virus antibodies in a population of students of tertiary institution in Nigeria | Full Text | Nigeria | Cross-sectional | Student | None Probability | 2003 | ELISA/EIA | Poor |
| Japhet 2019 | HIV, HBV AND HCV PREVALENCE, CO-INFECTIONS, RISK FACTORS AND AWARENESS AMONG STUDENTS IN A NIGERIAN UNIVERSITY | Abstract | Nigeria | Cross-sectional | Student | Unspecified | 2019 | ELISA/EIA | Moderate |
| Nnadozie 2021 | Serosurvey, awareness and sensitization on HIV, HBV, HCV and Mycobacterium tuberculosis among community-based population in Mbano, Imo State, Nigeria | Abstract | Nigeria | Cross-sectional | Mixed | None Probability | 2021 | RDT | Poor |
| Muhibi 2019 | Surveillance of anti-HCV antibody amongst in-school youth in a Nigeria university | Full Text | Nigeria | Cross-sectional | Student | None Probability | 2019 | ELISA/EIA | Moderate |
| Ogefere 2016 | Potential risk factors and seroprevalence of hepatitis c virus infection among students in a tertiary institution in Southern Nigeria | Full Text | Nigeria | Cross-sectional | Student | None Probability | 2016 | ELISA/EIA | Poor |
| Odukoya 2022 | Hepatitis B and C seroprevalence among residents in Lagos State, Nigeria: A population-based survey | Full Text | Nigeria | Cross-sectional | Mixed | Probability | 2014 | ELISA/EIA | High |
| Aliyu 2021 | The Burden of HIV, Hepatitis B and Hepatitis C by Armed Conflict Setting: The Nigeria AIDS Indicator and Impact Survey, 2018 | Full Text | Nigeria | Cross-sectional | Mixed | Probability | 2018 | RDT | High |
| Adda 2015 | Viral Hepatitis B & C epidemiology, disease burden, prevention and control measures in Taraba state | Abstract | Nigeria | Cross-sectional | Children | Probability | 2015 | Unspecified | Poor |
| Malu 2020 | Prevalence of Hepatitis B Surface Antigen and Antibodies to Hepatitis C in the General Population of Benue State, Central Nigeria | Full Text | Nigeria | Cross-sectional | Mixed | Probability | 2016 | RDT | High |
| Sule 2014 | Comparative burden of Hepatitis B and C virus infections to HIV among residents in North-Central Nigeria | Abstract | Nigeria | Cross-sectional | Mixed | Unspecified | 2012 | RDT | Poor |
| Ramyil 2014 | Prevalence and coinfection of human immunodeficiency virus, hepatitis B virus and hepatitis c virus in mabudi rural community of Plateau, State, Nigeria | Full Text | Nigeria | Cross-sectional | Mixed | None Probability | 2014 | RDT | Poor |
| Olal 2012 | Are patients with primary hepatocellular carcinoma infectious of hepatitis B, C and D viruses? | Full Text | Nigeria | Case-Control | Adult | Unspecified | 2012 | ELISA/EIA | Poor |
| Njoku 2016 | An Evaluation of Selected Populations for HIV-1 Vaccine Cohort Development in Nigeria | Full Text | Nigeria | Cross-sectional | Adult | Probability | 2009 | ELISA/EIA | Moderate |
| Quesada 2015 | Hepatitis C virus seroprevalence in the general female population from 8 countries | Full Text | Nigeria | Cross-sectional | Adult Women | Probability | 2005 | ELISA/EIA | High |
| Onyekwere 2015 | Hepatitis B and C virus prevalence and association with demographics: report of population screening in Nigeria | Full Text | Nigeria | Cross-sectional | Mixed | None Probability | 2012 | ELISA/EIA | High |
| Forbi 2012 | Epidemic history of hepatitis C virus infection in two remote communities in Nigeria, West Africa | Full Text | Nigeria | Cross-sectional | Mixed | Probability | 2007 | RDT & RNA-PCR | Poor |
| Forbi 2010 | Urban-rural estimation of hepatitis C virus infection sero-prevalence in north Central Nigeria | Short report | Nigeria | Cross-sectional | Mixed | Unspecified | 2007 | ELISA/EIA | Poor |
| Forbi 2009 | Serological markers and risk factors for hepatitis B and hepatitis C viruses among students in a Nigerian university | Full Text | Nigeria | Cross-sectional | Student | Probability | 2008 | ELISA/EIA | High |
| Nwankiti 2009 | Hepatitis C Virus infection in apparentenly healthy individuals with family history of diabetes in Vom, Plateau State Nigeria | Full Text | Nigeria | Cross-sectional | Mixed | None Probability | 2009 | ELISA/EIA | Poor |
| Ejele 2006 | Seroprevalence of hepatitis C virus in the Niger Delta of Nigeria | Abstract | Nigeria | Cross-sectional | Mixed | None Probability | 2006 | Unspecified | Poor |
| Eke 2016 | Seroprevalence and correlates of hepatitis c virus infection in secondary school children in Enugu, Nigeria | Full Text | Nigeria | Cross-sectional | Student | Probability | 2014 | ELISA/EIA | High |
| Olalekan 2015 | Prevalence and risk factors for hepatitis B and C among sexually active undergraduates in southwestern Nigeria | Full Text | Nigeria | Cross-sectional | Student | Probability | 2015 | Unspecified | Moderate |
| Okonkwo 2017 | Prevalence of hepatitis B, hepatitis C and human immunodeficiency viruses, and evaluation of risk factors for transmission: Report of a population screening in Nigeria | Full Text | Nigeria | Cross-sectional | Mixed | Probability | 2016 | ELISA/EIA | High |
| Jeremiah 2009 | Seroepidemiology of Transfusion Transmissible Viral Infection among University Fresh Students in Port Harcourt, Nigeria | Full Text | Nigeria | Cross-sectional | Student | Probability | 2008 | RDT | Moderate |
| Ugah 2021 | Epidemiology of hepatitis b virus, hepatitis C virus and human immunodeficiency virus co-infection among assymptomatic persons resident in Alex Ekwueme federal university Ndufu-alike | Full Text | Nigeria | Cross-sectional | Mixed | None Probability | 2021 | ELISA/EIA | Poor |
| Omolade 2018 | Prevalence of hepatitis C virus antibody among university students in Nigeria | Short report | Nigeria | Cross-sectional | Student | None Probability | 2018 | RDT | Poor |
| Pennap 2010 | Prevalence of hepatitis B and C virus infection among people of a local community in Keffi, Nigeria | Full Text | Nigeria | Cross-sectional | Mixed | None Probability | 2010 | ELISA/EIA | Moderate |
| Alkali 2023 | SEROEPIDEMIOLOGY OF HBV ANTIGEN AND ANTI-HCV AMONG GENERAL POPULATION IN A RURAL LOCAL IN BAUCHI STATE, NIGERIA | Abstract | Nigeria | Cross-sectional | Mixed group | Unclear | Unknown (2023) | RDT | Low |
|  |  |  | **Central** |  |  |  |  |  |  |
| Kamdem 2019 | Negative Association of Interleukin-33 Plasma Levels and Schistosomiasis Infection in a Site of Polyparasitism in Rural Cameroon | Full Text | Cameroon | Cross-sectional | Student | None Probability | 2018 | RDT | Moderate |
| Rodgers 2018 | Hepatitis C virus and human pegivirus 2 surveillance in a large cameroonian cohort | Abstract | Cameroon | Cross-sectional | Mixed | None Probability | 2016 | PCR | Moderate |
| Tazinkeng 2022 | Prevalence of and factors associated with hepatitis b and c in an urban health district in Cameroon, West Africa: A cross-sectional study OR Factors associated with hepatitis B and C among adults in Buea, Cameroon: A community-based cross-sectional study | Full Text | Cameroon | Cross-sectional | Mixed | None Probability | 2021 | RDT | High |
| Agbor 2018 | Prevalence of anti-hepatitis C antibodies and its co-infection with HIV in rural Cameroon | Full Text | Cameroon | Cross-sectional | Mixed | None Probability | 2015 | ELISA/EIA | Moderate |
| Njouom 2018 | Prevalence of hepatitis B, hepatitis C and hepatitis D virus infections in Cameroon: Results from a national population based survey (The ANRS 12289 project) OR  The burden of hepatitis C virus in Cameroon: Spatial epidemiology and historical perspective OR  The epidemiology of hepatitis delta virus infection in Cameroon | Full Text | Cameroon | Cross-sectional | Adult | Probability | 2011 | ELISA/EIA | High |
| Nansseu 2017 | Sero-epidemiology of human immunodeficiency virus, hepatitis B virus and hepatitis C virus: a cross-sectional survey in a rural setting of the West region of Cameroon | Full Text | Cameroon | Cross-sectional | Mixed | None Probability | 2014 | RDT | High |
| Mbopi-Keou 2015 | [Prevalence and factors associated with HIV and viral hepatitis B and C in the city of Bafoussam in Cameroon] | Full Text | Cameroon | Cross-sectional | Mixed | None Probability | 2012 | Unspecified | Poor |
| Foupouapouognigni 2011 | Hepatitis B and C virus infections in the three Pygmy groups in Cameroon | Full Text | Cameroon | Cross-sectional | Mixed | Unspecified | 2008 | ELISA & RNA-PCR | High |
| Pepin 2010 | Human parvovirus 4 infection, Cameroon  OR  Risk factors for hepatitis C virus transmission in colonial Cameroon | Full Text | Cameroon | Cross-sectional | Old | None Probability | 2009 | ELISA & RNA-PCR | Moderate |
| Nerrienet 2005 | Hepatitis C virus infection in cameroon: A cohort-effect | Full Text | Cameroon | Cross-sectional | Mixed | Unspecified | 1997 | ELISA/EIA | High |
| Njouom 2003 | High rate of hepatitis C virus infection and predominance of genotype 4 among elderly inhabitants of a remote village of the rain forest of South Cameroon | Full Text | Cameroon | Cross-sectional | Old | Probability | 1998 | ELISA & RNA-PCR | High |
| Kowo 1995 | Prevalence of hepatitis C virus and other blood-borne viruses in Pygmies and neighbouring Bantus in southern Cameroon | Full Text | Cameroon | Cross-sectional | Mixed | None Probability | 1994 | ELISA/EIA | Moderate |
| Delaporte 1994 | Hepatitis C in remote populations of southern Cameroon | Short report | Cameroon | Cross-sectional | Mixed | Unspecified | 1984 | ELISA/EIA | High |
| Louis 1994 | High prevalence of anti-hepatitis C virus antibodies in a Cameroon rural forest area | Full Text | Cameroon | Cross-sectional | Mixed | Probability | 1992 | ELISA/EIA | Moderate |
| Ndumbe 1993 | Infections among pygmies in the Eastern Province of Cameroon | Full Text | Cameroon | Cross-sectional | Adult | Probability | 1993 | ELISA/EIA | Moderate |
| Ngatchu 1992 | Seroprevalence of anti-HCV in an urban child population: a pilot survey in a developing area, Cameroon | Full Text | Cameroon | Cross-sectional | Student | Probability | 1992 | ELISA/EIA | High |
| Mencarini 1991 | Prevalence of anti-HCV antibodies in Cameroon | Short report | Cameroon | Cross-sectional | Adult | Unspecified | 1990 | ELISA/EIA | Poor |
| Biwole-Sida 2015 | Carriage prevalence of HCV markers in the working environment (Cameroun) | Full Text | Cameroon | Cross-sectional | Adult | None Probability | 2014 | RDT | Moderate |
| Fretz 1995 | HCV infection in a rural population of the Central African Republic (CAR): evidence for three additional subtypes of genotype 4. | Full Text | Central Africa Republic | Cross-sectional | Mixed | Probability | 1992 | ELISA & RNA-PCR | High |
| Pepin 2010 | Iatrogenic transmission of human T cell lymphotropic virus type 1 and hepatitis C virus through parenteral treatment and chemoprophylaxis of sleeping sickness in colonial Equatorial Africa | Full Text | Central Africa Republic | Cross-sectional | Old | None Probability | 2006 | ELISA & RNA-PCR | Moderate |
| Basimane-Bisimwa 2023 | Seroprevalence and molecular characterization of viral hepatitis and HIV co-infection in the Central African Republic | Full Text | Central Africa Republic | Cross-sectional | Mixed | Probability | 2010 | ELISA/EIA | High |
| Parr 2018 | Dried blood spots allow for efficient, largescale surveys of hepatitis c viremia OR  An Efficient, Large-Scale Survey of Hepatitis C Viremia in the Democratic Republic of the Congo Using Dried Blood Spots | Full Text | DR Congo | Cross-sectional | Adult | None Probability | 2014 | ELISA & RNA-PCR | Moderate |
| Hogan 2016 | Epidemic History and Iatrogenic Transmission of Blood-borne Viruses in Mid-20th Century Kinshasa | Full Text | DR Congo | Cross-sectional | Old | None Probability | 2012 | ELISA & RNA-PCR | Moderate |
| Cantaloube 2010 | Analysis of hepatitis C virus strains circulating in Republic of the Congo | Full Text | Republic of the Congo | Cross-sectional | Mixed | Probability | 2005 | ELISA & RNA-PCR | Moderate |
| Lopez 2019 | Prevalence of hepatitis b and HIV infections among healthy volunteers to participate in a pfSPZ vaccine clinical trial in equatorial guinea | Abstract | Equ Guinea | Cross-sectional | Adult | None Probability | 2019 | Unspecified | Poor |
| Owono 2019 | Screening failures: How to improve health promotion and coverage of vaccination? | Abstract | Equ Guinea | Cross-sectional | Mixed | None Probability | 2019 | Unspecified | Poor |
| Basaras 1999 | Seroprevalence of hepatitis B and C, and human immunodeficiency type 1 viruses in a rural population from the Republic of Equatorial Guinea | Full Text | Equ Guinea | Cross-sectional | Mixed | Unspecified | 1999 | ELISA/EIA | High |
| Siawaya 2015 | HIV, HBS, HCV, HTLV and syphilis prevalence among high school learners and university student in Libreville, Gabon | Short report | Gabon | Cross-sectional | Student | None Probability | 2014 | ELISA/EIA | Poor |
| Njouom 2012 | Phylogeography, risk factors and genetic history of hepatitis C virus in Gabon, central Africa | Full Text | Gabon | Cross-sectional | Mixed | Probability | 2008 | ELISA & RNA-PCR | High |
| Ndong-Atome 2008 | Hepatitis C virus infection may lead to slower emergence of P. falciparum in blood | Full Text | Gabon | Cross-sectional | Mixed | Probability | 2003 | ELISA & RNA-PCR | High |
| Richard-Lenoble 1995 | Hepatitis B, C, D, and E markers in rural equatorial African villages (Gabon) | Full Text | Gabon | Cross-sectional | Mixed | Probability | 1992 | ELISA/EIA | Poor |
| Delaporte 1993 | High level of hepatitis C endemicity in Gabon, equatorial Africa | Full Text | Gabon | Cross-sectional | Mixed | None Probability | 1993 | ELISA & RNA-PCR | High |
|  |  |  | **Eastern** |  |  |  |  |  |  |
| Ghebrekidan 1998 | Prevalence of infection with HIV, hepatitis B and C viruses, in four high risk groups in Eritrea | Full Text | Eritrea | Cross-sectional | Mixed | Unspecified | 1995 | ELISA/EIA | Poor |
| Taye 2019 | Prevalence and associated risk factors of Hepatitis B and C virus infections among mothers in Jimma, South West Ethiopia: a community-based study | Full Text | Ethiopia | Cross-sectional | Adult Women | Probability | 2016 | ELISA/EIA | Moderate |
| Abebe 2019 | Seroprevalence of Hepatitis B virus surface antigen, anti-hepatitis C virus antibody and their associated factors among mothers living in Harar, Eastern Ethiopia | Full Text | Ethiopia | Cross-sectional | Adult Women | Probability | 2017 | ELISA/EIA | High |
| Biazin 2019 | Determining seroprevalence of Hepatitis B and C virus infections and associated risk factors among apparently healthy mothers in Addis Ababa, Ethiopia | Full Text | Ethiopia | Cross-sectional | Adult Women | Probability | 2017 | ELISA/EIA | High |
| Woldegiorgis 2019 | Community-based sero-prevalence of hepatitis B and C infections in South Omo Zone, Southern Ethiopia | Full Text | Ethiopia | Cross-sectional | Mixed | Probability | 2018 | RDT | High |
| Abera 2017 | Community seroprevalence of hepatitis B, C and human immunodeficiency virus in adult population in gojjam zones, northwest Ethiopia | Full Text | Ethiopia | Cross-sectional | Adult | Probability | 2015 | RDT | High |
| Berhe 2007 | Intensity of Schistosoma mansoni, hepatitis B, age, and sex predict levels of hepatic periportal thickening/fibrosis (PPT/F): a large-scale community-based study in Ethiopia | Full Text | Ethiopia | Cross-sectional | Mixed | Probability | 2002 | ELISA/EIA | High |
| Ayele 2002 | Higher prevalence of anti-HCV antibodies among HIV-positive compared to HIV-negative inhabitants of Addis Ababa, Ethiopia | Full Text | Ethiopia | Cross-sectional | Mixed | Probability | 1994 | ELISA/EIA | High |
| Frommel 1993 | A survey of antibodies to hepatitis C virus in Ethiopia | Full Text | Ethiopia | Cross-sectional | Mixed | Unspecified | 1991 | ELISA/EIA | Poor |
| Sentjens 2000 | Risk factors for HCV, HTLV-I/II and HIV infection in various population subsets in Ethiopia | Abstract | Ethiopia | Cross-sectional | Adult | Unspecified | 2000 | ELISA/EIA | Poor |
| Kumalo 2022 | Undiagnosed Seroprevalence of Hepatitis B and C Virus Infections in the Community of Wolaita Zone, Southern Ethiopia | Full Text | Ethiopia | Cross-sectional | Mixed | Probability | 2021 | RDT | Moderate |
| Beykaso 2021 | Burden and Transmission Risks of Viral Hepatitis in Southern Ethiopia: Evidence Needed for Prevention and Control Measures | Full Text | Ethiopia | Cross-sectional | Mixed | Probability | 2020 | ELISA/EIA | High |
| Ntagirabiri 2014 | Prevalence of hepatitis C virus in Burundi: A nationwide survey | Full Text | Burundi | Cross-sectional | Mixed | Probability | 2002 | ELISA/EIA | High |
| Brouard 2022 | Hepatitis B, C, and delta in the general population in Mayotte: hepatitis B as a major public health concern | Full Text | Mayotte | Cross-sectional | Mixed | Probability | 2019 | ELISA & RNA-PCR | High |
| Kotikot 2016 | Reproductive tract infections among low risk women attending KAVI-VZV 001 Study in Nairobi, Kenya | Abstract | Kenya | Cross-sectional | Adult Women | None Probability | 2016 | Unspecified | Poor |
| Ly 2018 | The Prevalence of Hepatitis C Virus Antibody in HIV-Negative Persons in Kenya, 2007 | Full Text | Kenya | Cross-sectional | Adult | Probability | 2007 | ELISA & RNA-PCR | High |
| Kerubo 2015 | Hepatitis B, Hepatitis C and HIV-1 Coinfection in Two Informal Urban Settlements in Nairobi, Kenya | Full Text | Kenya | Cross-sectional | Adult | None Probability | 2007 | RDT | High |
| Ramarokoto 2008 | Seroprevalence of hepatitis C and associated risk factors in urban areas of Antananarivo, Madagascar OR  Hepatitis C virus infection and genotypes in Antananarivo, Madagascar | Full Text | Madagascar | Cross-sectional | Mixed | Probability | 2004 | ELISA & RNA-PCR | High |
| Morvan 1994 | Anti-HCV antibody prevalence among an asymptomatic population living in two villages in Madagascar | Short report | Madagascar | Cross-sectional | Mixed | Probability | 1993 | ELISA/EIA | Moderate |
| Zeller 1997 | [Prevalence of hepatitis C virus infection in the general population of Madagascar] | Full Text | Madagascar | Cross-sectional | Mixed | Probability | 1994 | ELISA/EIA | High |
| Muro 2013 | Seroprevalence of hepatitis B and C viruses among children in Kilimanjaro Region, Tanzania | Full Text | Tanzania | Cross-sectional | Children | Probability | 2008 | ELISA/EIA | Moderate |
| Mazigo 2017 | Co-infection of Schistosoma mansoni/hepatitis C virus and their associated factors among adult individuals living in fishing villages, north-western Tanzania | Full Text | Tanzania | Cross-sectional | Adult | Probability | 2012 | RDT | High |
| Puato 2007 | Does HCV infection have a more favourable outcome in Tanzanian people?. Data from the Lugalawa study | Full Text | Tanzania | Cross-sectional | Mixed | Unspecified | 2007 | ELISA & RNA-PCR | Moderate |
| Stark 2000 | Seroepidemiology of TT virus, GBC-C/HGV, and hepatitis viruses B, C, and E among women in a rural area of Tanzania | Full Text | Tanzania | Cross-sectional | Adult Women | Probability | 1996 | ELISA/EIA | Moderate |
| Tess 2000 | Seroprevalence of hepatitis C virus in the general population of northwest Tanzania | Full Text | Tanzania | Cross-sectional | Adult | Probability | 1990 | ELISA & RNA-PCR | High |
| Miller 1998 | Seroprevalence of viral hepatitis in Tanzanian adults | Full Text | Tanzania | Cross-sectional | Adult | None Probability | 1992 | ELISA/EIA | Moderate |
| Ilardi 1992 | Prevalence of anti-HCV in two Tanzanian villages | Abstract | Tanzania | Cross-sectional | Mixed | Probability | 1992 | ELISA/EIA | Poor |
| Laukmamm-Josten 1993 | HIV, hepatitis B and C seroprevalence in truckstops and near by communities in Tanzania | Abstract | Tanzania | Cross-sectional | Mixed | None Probability | 1993 | ELISA/EIA | Poor |
| O'Hara 2020 | Liver function tests and fibrosis scores in a rural population in Africa: a cross-sectional study to estimate the burden of disease and associated risk factors | Full Text | Uganda | Cross-sectional | Mixed | Probability | 2011 | ELISA & RNA-PCR | High |
| Davis 2019 | Highly Diverse Hepatitis C Strains Detected in Sub-Saharan Africa Have Unknown Susceptibility to Direct-Acting Antiviral Treatments | Full Text | Uganda | Cross-sectional | Mixed | Probability | 2011 | ELISA & RNA-PCR | High |
| Mullis 2013 | High frequency of false-positive hepatitis C virus enzyme-linked immunosorbent assay in Rakai, Uganda | Full Text | Uganda | Case-Control | Mixed | None Probability | 2013 | ELISA & RNA-PCR | High |
| Makuza 2020 | Role of unsafe medical practices and sexual behaviours in the hepatitis B and C syndemic and HIV co-infection in Rwanda: a cross-sectional study | Full Text | Rwanda | Cross-sectional | Mixed | None Probability | 2019 | RDT | High |
| Iradukunda 2020 | Risk factors associated with hepatitis B and C in rural population of Burera district, Rwanda | Full Text | Rwanda | Cross-sectional | Mixed | None Probability | 2017 | ELISA/EIA | Poor |
| Makuza 2019 | Risk factors for viral hepatitis C infection in Rwanda: results from a nationwide screening program | Full Text | Rwanda | Cross-sectional | Mixed | None Probability | 2018 | ELISA/EIA | High |
| Umutesi 2019 | Screening a nation for hepatitis C virus elimination: a cross-sectional study on prevalence of hepatitis C and associated risk factors in the Rwandan general population | Full Text | Rwanda | Cross-sectional | Mixed | None Probability | 2017 | ELISA & RNA-PCR | High |
| Kamali 2022 | Time to complete hepatitis C cascade of care among patients identified during mass screening campaigns in rural Rwanda: a retrospective cohort study | Full Text | Rwanda | Secondary data analysis of household survey | Mixed | Probability | 2019 | RDT & RNA-PCR | Moderate |
| Nisingizwe 2023 | The Cascade of Care for Hepatitis C Treatment in Rwanda: A Retrospective Cohort Study of the 2017–2019 Mass Screening and Treatment Campaign | Full Text | Rwanda | Secondary data analysis of household survey | Mixed | Probability | 2019 | ELISA & RNA-PCR | High |
| Bovet 1999 | Decrease in the prevalence of hepatitis B and a low prevalence of hepatitis C virus infections in the general population of the Seychelles | Full Text | Seychelles | Cross-sectional | Adult | Probability | 1994 | ELISA/EIA | High |
| Bile 1992 | The risk for hepatitis A, B, and C at two institutions for children in Somalia with different socioeconomic conditions | Abstract | Somalia | Cross-sectional | Children | Unspecified | 1992 | Unspecified | Poor |
| Sube 2020 | Prevalence of HBV, HCV, HIV and syphilis infections among secondary school students in Juba, South Sudan | Abstract | South Sudan | Cross-sectional | Student | Unspecified | 2017 | Unspecified | Poor |
| Mudawi 2007 | Epidemiology of HCV infection in Gezira state of central Sudan OR  Prevalence of HCV infection in an endemic area for schistosomal infection and in patients with hepatosplenic schistosomiasis in Sudan | Full Text | Sudan | Cross-sectional | Mixed | Probability | 2000 | ELISA & RNA-PCR | Moderate |
| Omer 2001 | The role of hepatitis B and hepatitis C viral infections in the incidence of hepatocellular carcinoma in Sudan | Full Text | Sudan | Case-Control | Mixed | Probability | 1998 | ELISA/EIA | High |
| Froeschl 2021 | Hepatitis B, C and D virus prevalence in children and adults in Mbeya Region, Tanzania: results from a cohort study 2002 - 2009 | Full Text | Tanzania | Cross-sectional | Mixed | None Probability | 2011 | ELISA/EIA | Moderate |
|  |  |  | **Southern** |  |  |  |  |  |  |
| Stockdale 2022 | A clinical and molecular epidemiological survey of hepatitis C in Blantyre, Malawi, suggests a historic mechanism of transmission | Full Text | Malawi | Cross-sectional | Mixed | Probability | 2018 | ELISA & RNA-PCR | Moderate |
| Taha 2015 | Seroprevalence for Hepatitis E and Other Viral Hepatitides among Diverse Populations, Malawi | Full Text | Malawi | Cross-sectional | Mixed | Unspecified | 2001 | ELISA/EIA | Poor |
| Fox 2015 | Prevalence of hepatitis C virus in mothers and their children in Malawi | Full Text | Malawi | Cross-sectional | Adult Women | None Probability | 2010 | ELISA/EIA | Moderate |
| AbdoolKarim 1993 | Hepatitis C virus infection in urban and rural Natal/KwaZulu | Full Text | South Africa | Cross-sectional | Mixed | Probability | 1985 | ELISA/EIA | Moderate |
|  |  |  |  |  |  |  |  |  |  |
| Kallestrup 2003 | Low prevalence of hepatitis C virus antibodies in HIV-endemic area of Zimbabwe support sexual transmission as the major route of HIV transmission in Africa | Abstract | Zimbabwe | Cross-sectional | Mixed | None Probability | 2003 | ELISA/EIA | Poor |
|  |  |  | Multiple Regions/Countries |  |  |  |  |  |  |
| Stevens 2008 | Baseline morbidity in 2,990 adult African volunteers recruited to characterize laboratory reference intervals for future HIV vaccine clinical trials | Full Text | Kenya/Uganda/Zambia | Cross-sectional | Adult | None Probability | 2006 | ELISA/EIA | Moderate |
| Coursaget 1990 | Prevalence of hepatitis-c virus-infection in africa - anti-hcv antibodies in the general-population and in patients suffering from cirrhosis or primary liver-cancer | Full Text | Burundi/Madagascar/Senegal | Cross-sectional | Adult | None Probability | 1986 | ELISA/EIA | Poor |
| Jeannel 1998 | Evidence for high genetic diversity and long-term endemicity of hepatitis C virus genotypes 1 and 2 in West Africa | Full Text | Burkina Faso/Guinea | Cross-sectional | Mixed | Probability | 1995 | ELISA & RNA-PCR | Moderate |

Mixed population: studies done in all age groups and box sex; JBI: Joanna Briggs Institute; ELISA: Enzyme Linked Immunosorbent Assay/Enzyme Immune Assay (ELISA/EIA); RNA: ribonucleic acid.

1. **DESTINE NIHR GHR Group members.**

Professor John F Dillon (University of Dundee), Professor Wondwossen Amogne Degu (Addis Ababa University), Professor Peter Vickerman (University of Bristol), Professor Matthew Hickman (University of Bristol), Professor Ora Paltiel (Hadassah-Hebrew University), Dr Dawit Wolday (Ethiopian Public Health Institute/ McMaster University), Dr Aynishet Adane (University of Gondar), Mrs Saro Abdella (University of Dundee/Ethiopian Public Health Institute), Dr Zenahbezu Abay (University of Gondar), Dr Workagegnehu Hailu (University of Gondar), Professor Tadesse Awoke (University of Gondar), Dr Emebet Dagne (Jimma University), Dr Elias Ali Yesuf (Jimma University), Dr Josephine G Walker (University of Bristol), Dr Aaron G Lim (University of Bristol), Dr Clare E French (University of Bristol), Dr Andaragachew Mulu (Armauer Hansen Research Institute), Melaku Tileku Tamiru (University of Dundee/Addis Ababa University), Atsbeha Gebreegziabxier Weldemariam (University of Dundee/Ethiopian Public Health Institute), Dr Christie Cabral (University of Bristol), Dr Obsie Baissa (Hadassah-Hebrew University), Ms Elizabeth Speakman (University of Dundee), Dr Andrew Radley (NHS Tayside), Dr Amy Malaguti (NHS Tayside), Dr Sarah Inglis (University of Dundee), Ms Meseret Yohannes (Addis Ababa University), Ms Bruktait Taddele (Addis Ababa University), Dr Hagos Abraha (Mekelle University), Dr Mengistu Erkie (Addis Ababa University), Tesfa Sewunet Alamneh (University of Bristol/University of Gondar), Getahun Molla Kassa (University of Bristol/University of Gondar).

**Roles of the funding source:** This research was funded by the NIHR (NIHR133208) using UK international development funding from the UK Government to support global health research. The views expressed in this publication are those of the author(s) and not necessarily those of the NIHR or the UK Department of Health and Social Care.

1. **Bibliography (3-141)**

1. Munn Z, Moola S, Lisy K, Riitano D, Tufanaru C. Methodological guidance for systematic reviews of observational epidemiological studies reporting prevalence and cumulative incidence data. Int J Evid Based Healthc. 2015;13(3):147-53.

2. World Health Organization African Region. Hepatitis Scorecard for the WHO Africa Region Implementing the hepatitis elimination strategy 2023 [cited 2023 January 12]. Available from: <https://www.afro.who.int/publications/hepatitis-scorecard-who-africa-region-implementing-hepatitis-elimination-strategy>.

3. Abdool Karim SS, Tait DR. Hepatitis C virus infection in urban and rural Natal/KwaZulu. South African medical journal = Suid-Afrikaanse tydskrif vir geneeskunde. 1993;83(3):191-3.

4. Abebe F, Seyoum B, Teklemariam Z, Oljira L, Tarekegne A, Bekele F, et al. Seroprevalence of Hepatitis B virus surface antigen, anti-hepatitis C virus antibody and their associated factors among mothers living in Harar, Eastern Ethiopia. Ethiop med j (Online). 2019;57(3):119-27.

5. Abera B, Adem Y, Yimer M, Mulu W, Zenebe Y, Mekonnen Z. Community seroprevalence of hepatitis B, C and human immunodeficiency virus in adult population in gojjam zones, northwest Ethiopia. VIROLOGY JOURNAL. 2017;14(1):21.

6. Adda DK. Viral Hepatitis B & C epidemiology, disease burden, prevention and control measures in Taraba state. JOURNAL OF VIRAL HEPATITIS. 2015;22:42-3.

7. Agbor VN, Tagny CT, Kenmegne J-B, Awazi B, Ngansop C, Mbanya D, et al. Prevalence of anti-hepatitis C antibodies and its co-infection with HIV in rural Cameroon. BMC RESEARCH NOTES. 2018;11(1):459.

8. Aliyu GG, Aliyu SH, Ehoche A, Dongarwar D, Yusuf RA, Aliyu MH, et al. The Burden of HIV, Hepatitis B and Hepatitis C by Armed Conflict Setting: The Nigeria AIDS Indicator and Impact Survey, 2018. ANNALS OF GLOBAL HEALTH. 2021;87(1):53.

9. Alkali M, Okon KO, Umar MS, Sani MD, Shuaibu H, Babale RS, et al. SEROEPIDEMIOLOGY OF HBV ANTIGEN AND ANTI-HCV AMONG GENERAL POPULATION IN A RURAL LOCAL IN BAUCHI STATE, NIGERIA. West African journal of medicine. 2023(12 Suppl 1):S33-S4.

10. Ayele W, Nokes DJ, Abebe A, Messele T, Dejene A, Enquselassie F, et al. Higher prevalence of anti-HCV antibodies among HIV-positive compared to HIV-negative inhabitants of Addis Ababa, Ethiopia. JOURNAL OF MEDICAL VIROLOGY. 2002;68(1):12-7.

11. Basaras M, Santamaria A, Sarsa M, Gutierrez E, de Olano Y, Cisterna R. Seroprevalence of hepatitis B and C, and human immunodeficiency type 1 viruses in a rural population from the Republic of Equatorial Guinea. TRANSACTIONS OF THE ROYAL SOCIETY OF TROPICAL MEDICINE AND HYGIENE. 1999;93(3):250-2.

12. Basimane-Bisimwa P, Koyaweda GW, Ngaiganam E, Vickos U, Sibiro OAD, Yambiyo BM, et al. Seroprevalence and molecular characterization of viral hepatitis and HIV co-infection in the Central African Republic. medRxiv. 2023.

13. Berhe N, Myrvang B, Gundersen SG. Intensity of Schistosoma mansoni, hepatitis B, age, and sex predict levels of hepatic periportal thickening/fibrosis (PPT/F): a large-scale community-based study in Ethiopia. The American journal of tropical medicine and hygiene. 2007;77(6):1079-86.

14. Beykaso G, Mulu A, Giday M, Berhe N, Selamu M, Mihret A, et al. Burden and Transmission Risks of Viral Hepatitis in Southern Ethiopia: Evidence Needed for Prevention and Control Measures. RISK MANAGEMENT AND HEALTHCARE POLICY. 2021;14:4843-52.

15. Biazin H, Teshome S, Ayenew Z, Abebe T, Mihret A, Aseffa A, et al. Determining seroprevalence of Hepatitis B and C virus infections and associated risk factors among apparently healthy mothers in Addis Ababa, Ethiopia. Ethiop med j (Online). 2019;57(3):129-38.

16. Bile K, Mohamud O, Aden C, Isse A, Norder H, Nilsson L, et al. The risk for hepatitis A, B, and C at two institutions for children in Somalia with different socioeconomic conditions. The American journal of tropical medicine and hygiene. 1992;47(3):357-64.

17. Biwole-Sida M, Noah D, Eloumou AF, Dang I, Talla P, Malongue, et al. Carriage prevalence of HCV markers in the working environment (Cameroun). JOURNAL AFRICAIN D HEPATO-GASTROENTEROLOGIE. 2015;9(1):26-9.

18. Bovet P, Yersin C, Herminie P, Lavanchy D, Frei PC. Decrease in the prevalence of hepatitis B and a low prevalence of hepatitis C virus infections in the general population of the Seychelles. BULLETIN OF THE WORLD HEALTH ORGANIZATION. 1999;77(11):923-8.

19. Brouard C, Parenton F, Youssouf H, Chevaliez S, Gordien E, Jean M, et al. Hepatitis B, C, and delta in the general population in Mayotte: hepatitis B as a major public health concern. BMC INFECTIOUS DISEASES. 2022;22(1):716.

20. Cantaloube J-F, Gallian P, Bokilo A, Jordier F, Biagini P, Attoui H, et al. Analysis of hepatitis C virus strains circulating in Republic of the Congo. JOURNAL OF MEDICAL VIROLOGY. 2010;82(4):562-7.

21. Clifford GM, Waterboer T, Dondog B, Qiao YL, Kordzaia D, Hammouda D, et al. Hepatitis C virus seroprevalence in the general female population of 9 countries in Europe, Asia and Africa. INFECTIOUS AGENTS AND CANCER. 2017;12.

22. Coursaget P, Bourdil C, Kastally R, Yvonnet B, Rampanarivo Z, Chiron JP, et al. PREVALENCE OF HEPATITIS-C VIRUS-INFECTION IN AFRICA - ANTI-HCV ANTIBODIES IN THE GENERAL-POPULATION AND IN PATIENTS SUFFERING FROM CIRRHOSIS OR PRIMARY LIVER-CANCER. RESEARCH IN VIROLOGY. 1990;141(4):449-54.

23. Davis C, Mgomella GS, da Silva Filipe A, Frost EH, Giroux G, Hughes J, et al. Highly Diverse Hepatitis C Strains Detected in Sub-Saharan Africa Have Unknown Susceptibility to Direct-Acting Antiviral Treatments. Hepatology (Baltimore, Md). 2019;69(4):1426-41.

24. Delaporte E, Froment A, Dazza MC, Henzel D, Larouze B. Hepatitis C in remote populations of southern Cameroon. ANNALS OF TROPICAL MEDICINE AND PARASITOLOGY. 1994;88(1):97-8.

25. Delaporte E, Thiers V, Dazza MC, Romeo R, Mlika-Cabanne N, Aptel I, et al. High level of hepatitis C endemicity in Gabon, equatorial Africa. TRANSACTIONS OF THE ROYAL SOCIETY OF TROPICAL MEDICINE AND HYGIENE. 1993;87(6):636-7.

26. Diallo MS, Sylla A, Sidibe K, Sylla BS, Trepo CR, Wild CP. Prevalence of exposure to aflatoxin and hepatitis B and C viruses in Guinea, West Africa. Natural toxins. 1995;3(1):6-9.

27. Ejele OA, Nwauche CA, Erhabor O. Seroprevalence of hepatitis C virus in the Niger Delta of Nigeria. The Nigerian postgraduate medical journal. 2006;13(2):103-6.

28. Eke CB, Ogbodo SO, Ukoha OM, Muoneke VU, Ibekwe RC, Ikefuna AN. Seroprevalence and correlates of hepatitis c virus infection in secondary school children in Enugu, Nigeria. ANNALS OF MEDICAL AND HEALTH SCIENCES RESEARCH. 2016;6(3):156-61.

29. Forbi J, Pennap G, Silas-Ndukuba C, Agabi Y, Agwale S. Serological markers and risk factors for hepatitis B and hepatitis C viruses among students in a Nigerian university. East African journal of public health. 2009;6(2):152-5.

30. Forbi JC, Pietzsch J, Olaleye VO, Forbi TD, Pennap GR, Esona MD, et al. Urban-rural estimation of hepatitis C virus infection sero-prevalence in north Central Nigeria. East African journal of public health. 2010;7(4):367-8.

31. Forbi JC, Purdy MA, Campo DS, Vaughan G, Dimitrova ZE, Ganova-Raeva LM, et al. Epidemic history of hepatitis C virus infection in two remote communities in Nigeria, West Africa. The Journal of general virology. 2012;93:1410-21.

32. Foupouapouognigni Y, Mba SAS, Betsem a Betsem E, Rousset D, Froment A, Gessain A, et al. Hepatitis B and C virus infections in the three Pygmy groups in Cameroon. JOURNAL OF CLINICAL MICROBIOLOGY. 2011;49(2):737-40.

33. Fox JM, Newton R, Bedaj M, Keding A, Molyneux E, Carpenter LM, et al. Prevalence of hepatitis C virus in mothers and their children in Malawi. TROPICAL MEDICINE & INTERNATIONAL HEALTH. 2015;20(5):638-42.

34. Fretz C, Jeannel D, Stuyver L, Herve V, Lunel F, Boudifa A, et al. HCV infection in a rural population of the Central African Republic (CAR): evidence for three additional subtypes of genotype 4. JOURNAL OF MEDICAL VIROLOGY. 1995;47(4):435-7.

35. Fretz C, Jeannel D, Stuyver L, Herve V, Lunel F, Boudifa A, et al. HCV INFECTION IN A RURAL-POPULATION OF THE CENTRAL-AFRICAN-REPUBLIC (CAR) - EVIDENCE FOR 3 ADDITIONAL SUBTYPES OF GENOTYPE-4. JOURNAL OF MEDICAL VIROLOGY. 1995;47(4):435-7.

36. Froeschl G, Hoelscher M, Maganga LH, Kroidl I, Clowes P, Geis S, et al. Hepatitis B, C and D virus prevalence in children and adults in Mbeya Region, Tanzania: results from a cohort study 2002 - 2009. The Pan African medical journal. 2021;39:174.

37. Frommel D, Tekle-Haimanot R, Berhe N, Aussel L, Verdier M, Preux PM, et al. A survey of antibodies to hepatitis C virus in Ethiopia. The American journal of tropical medicine and hygiene. 1993;49(4):435-9.

38. Ghebrekidan H, Cox S, Wahren B, Grandien M. Prevalence of infection with HIV, hepatitis B and C viruses, in four high risk groups in Eritrea. CLINICAL AND DIAGNOSTIC VIROLOGY. 1998;9(1):29-35.

39. Guindo CO, Katile A, Kamate B, Guindo MA, Zeguime A, Doucoure M, et al. Causes of screening failure during a transmission blocking vaccine trial around Doneguebougou, Mali. AMERICAN JOURNAL OF TROPICAL MEDICINE AND HYGIENE. 2018;99(4):137.

40. Guindo MA, Diakite ML, Ouologuem B, Traore S, Konate A, Katile A, et al. Hepatitis b, hepatitis c and HIV infection frequencies among volunteers screened for malaria vaccine clinical trials in Mali. AMERICAN JOURNAL OF TROPICAL MEDICINE AND HYGIENE. 2014;91(5):488.

41. Hodges M, Sanders E, Aitken C. Seroprevalence of hepatitis markers; HAV, HBV, HCV and HEV amongst primary school children in Freetown, Sierra Leone. West African journal of medicine. 1998;17(1):36-7.

42. Hogan CA, Iles J, Frost EH, Giroux G, Cassar O, Gessain A, et al. Epidemic History and Iatrogenic Transmission of Blood-borne Viruses in Mid-20th Century Kinshasa. The Journal of infectious diseases. 2016;214(3):353-60.

43. Honge BL, Olesen JS, Jensen MM, Jespersen S, da Silva ZJ, Rodrigues A, et al. Hepatitis B and C in the adult population of Bissau, Guinea-Bissau: a cross-sectional survey. Tropical medicine & international health : TM & IH. 2020;25(2):255-63.

44. Ilardi I, Errera G, De Sanctis GM, Barbacini IG, Madera A, Leone F, et al. Prevalence of anti-HCV in two Tanzanian villages. Archives of virology Supplementum. 1992;4:347-8.

45. Iradukunda PG, Habyarimana T, Niyonzima FN, Uwitonze A-Y, Mpunga T. Risk factors associated with hepatitis B and C in rural population of Burera district, Rwanda. The Pan African medical journal. 2020;35:43.

46. Japhet M, Adewumi M, Olufisayo A. HIV, HBV AND HCV PREVALENCE, CO-INFECTIONS, RISK FACTORS AND AWARENESS AMONG STUDENTS IN A NIGERIAN UNIVERSITY. BMJ GLOBAL HEALTH. 2019;4:A59.

47. Jeannel D, Fretz C, Traore Y, Kohdjo N, Bigot A, Pe Gamy E, et al. Evidence for high genetic diversity and long-term endemicity of hepatitis C virus genotypes 1 and 2 in West Africa. JOURNAL OF MEDICAL VIROLOGY. 1998;55(2):92-7.

48. Jemilohun AC, Oyelade BO, Oiwoh SO. Prevalence of Hepatitis C virus antibody among undergraduates in Ogbomosho, South-Western Nigeria. Afr j infect dis (Online). 2014;8(2):40-3.

49. Jeremiah ZA, Tony-Enwin EO. Seroepidemiology of Transfusion Transmissible Viral Infection among University Fresh Students in Port Harcourt, Nigeria. HEPATITIS MONTHLY. 2009;9(4):276-81.

50. Jolly PE, Jiang Y, Ellis WO, Awuah RT, Appawu J, Nnedu O, et al. Association between aflatoxin exposure and health characteristics, liver function, hepatitis and malaria infections in Ghanaians. Journal of Nutritional and Environmental Medicine. 2007;16(3):242-57.

51. Kalinina OV, Lichnaia EV, Boiro MY, Totolian AA. THE OCCURRENCE OF THE MARKERS OF HEPATITIS C AMONG PRACTICALLY HEALTHY RESIDENTS OF THE REPUBLIC OF GUINEA: A PILOT STUDY. INFEKTSIYA I IMMUNITET. 2017;7(3):245-50.

52. Kallestrup P, Zinyama R, Gomo E, Dickmeiss E, Platz P, Gerstoft J, et al. Low prevalence of hepatitis C virus antibodies in HIV-endemic area of Zimbabwe support sexual transmission as the major route of HIV transmission in Africa. AIDS. 2003;17(9):1400-2.

53. Kamali I, Shumbusho F, Barnhart DA, Nyirahabihirwe F, Gakuru JD, Dusingizimana W, et al. Time to complete hepatitis C cascade of care among patients identified during mass screening campaigns in rural Rwanda: a retrospective cohort study. BMC INFECTIOUS DISEASES. 2022;22(1).

54. Kamdem SD, Konhawa F, Kuemkon EM, Meyo Kamguia L, Tchanana GK, Nche F, et al. Negative Association of Interleukin-33 Plasma Levels and Schistosomiasis Infection in a Site of Polyparasitism in Rural Cameroon. FRONTIERS IN IMMUNOLOGY. 2019;10:2827.

55. Kerubo G, Khamadi S, Okoth V, Madise N, Ezeh A, Ziraba A, et al. Hepatitis B, Hepatitis C and HIV-1 Coinfection in Two Informal Urban Settlements in Nairobi, Kenya. PLOS ONE. 2015;10(6):e0129247.

56. Kotikot T, Ndalamia J, Ogutu H, Nyaoke B, Mureithi W, Farah B, et al. Reproductive tract infections among low risk women attending KAVI-VZV 001 Study in Nairobi, Kenya. AIDS RESEARCH AND HUMAN RETROVIRUSES. 2016;32:281.

57. Kowo MP, Goubau P, Ndam EC, Njoya O, Sasaki S, Seghers V, et al. Prevalence of hepatitis C virus and other blood-borne viruses in Pygmies and neighbouring Bantus in southern Cameroon. TRANSACTIONS OF THE ROYAL SOCIETY OF TROPICAL MEDICINE AND HYGIENE. 1995;89(5):484-6.

58. Kpossou AR, Kouwakanou B, Sokpon CNDM, Alassane KS, Bankole MM, Ahouada C, et al. [Seroprevalence of hepatitis C virus (HCV) antibodies and associated factors based on voluntary screening data collection in the general population in Benin in 2016]. Seroprevalence des anticorps anti-virus de l'hepatite C et facteurs associes, d'apres un depistage volontaire en population generale en 2016 au Benin. 2021;40:30.

59. Kpossou AR, Paraiso MN, Sokpon CNd, Alassan KS, Vignon RK, Keke RK, et al. [Seroprevalence of viral hepatitis B and its associated factors determined based on data from a screening campaign targeting the general population in Benin]. Hepatite virale B lors d'une campagne de depistage en population generale au Benin: seroprevalence et facteurs associes. 2020;37:247.

60. Kumalo A, Teklu T, Demisse T, Anjulo A. Undiagnosed Seroprevalence of Hepatitis B and C Virus Infections in the Community of Wolaita Zone, Southern Ethiopia. HEPATIC MEDICINE-EVIDENCE AND RESEARCH. 2022;14:111-22.

61. Laukmamm-Josten U, Ocheng D, Mwizarubi BK, Swai R, Nyamurekunge K. HIV, hepatitis B and C seroprevalence in truckstops and near by communities in Tanzania. Proceedings of the Ninth International Conference on AIDS/III. 1993.

62. Lavoie M, Sharp CP, Pepin J, Pennington C, Foupouapouognigni Y, Pybus OG, et al. Human parvovirus 4 infection, Cameroon. EMERGING INFECTIOUS DISEASES. 2012;18(4):680-3.

63. Lingani M, Akita T, Ouoba S, Nagashima S, Boua PR, Takahashi K, et al. The changing epidemiology of hepatitis B and C infections in Nanoro, rural Burkina Faso: a random sampling survey. BMC INFECTIOUS DISEASES. 2020;20(1):46.

64. Lopez MSA, Hamad A, Ramadhani K, Urbano V, Owono G, Lobede F, et al. Prevalence of hepatitis b and HIV infections among healthy volunteers to participate in a pfSPZ vaccine clinical trial in equatorial guinea. AMERICAN JOURNAL OF TROPICAL MEDICINE AND HYGIENE. 2019;101(5):324.

65. Louis FJ, Maubert B, Le Hesran JY, Kemmegne J, Delaporte E, Louis JP. High prevalence of anti-hepatitis C virus antibodies in a Cameroon rural forest area. TRANSACTIONS OF THE ROYAL SOCIETY OF TROPICAL MEDICINE AND HYGIENE. 1994;88(1):53-4.

66. Ly KN, Kim AA, Drobeniuc J, Kodani M, Montgomery JM, Fields BS, et al. The Prevalence of Hepatitis C Virus Antibody in HIV-Negative Persons in Kenya, 2007. The American journal of tropical medicine and hygiene. 2018;98(6):1876-9.

67. Mabayoje VO, Akinwusi PO, Opaleye O, Egbewale BE, Fagbami AH. Prevalence of hepatitis B surface antigen, hepatitis C and human immunodeficiency virus antibodies in a population of students of tertiary institution in Nigeria. Afr J Clin Exp Microbiol. 2010;11(2):68-74.

68. Makuza JD, Liu CY, Ntihabose CK, Dushimiyimana D, Umuraza S, Nisingizwe MP, et al. Risk factors for viral hepatitis C infection in Rwanda: results from a nationwide screening program. BMC INFECTIOUS DISEASES. 2019;19(1):688.

69. Makuza JD, Nisingizwe MP, Rwema JOT, Dushimiyimana D, Habimana DS, Umuraza S, et al. Role of unsafe medical practices and sexual behaviours in the hepatitis B and C syndemic and HIV co-infection in Rwanda: a cross-sectional study. BMJ OPEN. 2020;10(7):e036711.

70. Malu AO, Achinge GI, Utoo PM, Kur JT, Obekpa SA. Prevalence of Hepatitis B Surface Antigen and Antibodies to Hepatitis C in the General Population of Benue State, Central Nigeria. The American journal of tropical medicine and hygiene. 2020;102(5):995-1000.

71. Martinson FE, Weigle KA, Mushahwar IK, Weber DJ, Royce R, Lemon SM. Seroepidemiological survey of hepatitis B and C virus infections in Ghanaian children. JOURNAL OF MEDICAL VIROLOGY. 1996;48(3):278-83.

72. Mazigo HD, Kepha S, Kaatano GM, Kinung'hi SM. Co-infection of Schistosoma mansoni/hepatitis C virus and their associated factors among adult individuals living in fishing villages, north-western Tanzania. BMC INFECTIOUS DISEASES. 2017;17(1):668.

73. Mbopi-Keou F-X, Nkala IVM, Kalla GCM, Nguefack-Tsague G, Kamga HG, Noubom M, et al. [Prevalence and factors associated with HIV and viral hepatitis B and C in the city of Bafoussam in Cameroon]. Seroprevalence et facteurs associes au VIH et aux hepatites virales B et C dans la ville de Bafoussam au Cameroun. 2015;20:156.

74. Meda N, Tuaillon E, Kania D, Tiendrebeogo A, Pisoni A, Zida S, et al. Hepatitis B and C virus seroprevalence, Burkina Faso: a cross-sectional study. BULLETIN OF THE WORLD HEALTH ORGANIZATION. 2018;96(11):750-9.

75. Mencarini P, De Luca A, Antinori A, Maiuro G, Spedini G, Bailly C, et al. Prevalence of anti-HCV antibodies in Cameroon. TRANSACTIONS OF THE ROYAL SOCIETY OF TROPICAL MEDICINE AND HYGIENE. 1991;85(5):654-5.

76. Miller WC, Shao JF, Weaver DJ, Shimokura GH, Paul DA, Lallinger GJ. Seroprevalence of viral hepatitis in Tanzanian adults. Tropical medicine & international health : TM & IH. 1998;3(9):757-63.

77. Morvan JM, Boisier P, Roux JF. Anti-HCV antibody prevalence among an asymptomatic population living in two villages in Madagascar. TRANSACTIONS OF THE ROYAL SOCIETY OF TROPICAL MEDICINE AND HYGIENE. 1994;88(6):657.

78. Mudawi HMY, Smith HM, Rahoud SA, Fletcher IA, Babikir AM, Saeed OK, et al. Epidemiology of HCV infection in Gezira state of central Sudan. JOURNAL OF MEDICAL VIROLOGY. 2007;79(4):383-5.

79. Muhibi MA, Ifeanyichukwu MO, Olawuyi AO, Abulude AA, Adeyemo MO, Muhibi MO. Surveillance of anti-HCV antibody amongst in-school youth in a Nigeria university. African Journal of Clinical and Experimental Microbiology. 2019;20(1):49-53.

80. Mullis CE, Laeyendecker O, Reynolds SJ, Ocama P, Quinn J, Boaz I, et al. High frequency of false-positive hepatitis C virus enzyme-linked immunosorbent assay in Rakai, Uganda. Clinical infectious diseases : an official publication of the Infectious Diseases Society of America. 2013;57(12):1747-50.

81. Muro FJ, Fiorillo SP, Sakasaka P, Odhiambo C, Reddy EA, Cunningham CK, et al. Seroprevalence of hepatitis B and C viruses among children in Kilimanjaro Region, Tanzania. JOURNAL OF THE PEDIATRIC INFECTIOUS DISEASES SOCIETY. 2013;2(4):320-6.

82. Nansseu JR, Mbogning DM, Monamele GC, Tamoh SF, Gonsu HK, Kouanfack C, et al. Sero-epidemiology of human immunodeficiency virus, hepatitis B virus and hepatitis C virus: a cross-sectional survey in a rural setting of the West region of Cameroon. The Pan African medical journal. 2017;28:201.

83. Ndow G, Cohen D, Shimakawa Y, Gore ML, Njie R, Suso P, et al. Occult Hepatitis B infection is frequent and a risk factor of advanced liver disease in The Gambia, West Africa. JOURNAL OF HEPATOLOGY. 2018;68:S485-S6.

84. Ndumbe PM, Atchou G, Biwole M, Lobe V, Ayuk-Takem J. Infections among pygmies in the Eastern Province of Cameroon. Medical microbiology and immunology. 1993;182(6):281-4.

85. Nerrienet E, Pouillot R, Lachenal G, Njouom R, Mfoupouendoun J, Bilong C, et al. Hepatitis C virus infection in cameroon: A cohort-effect. JOURNAL OF MEDICAL VIROLOGY. 2005;76(2):208-14.

86. Ngatchu T, Stroffolini T, Rapicetta M, Chionne P, Lantum D, Chiaramonte M. Seroprevalence of anti-HCV in an urban child population: a pilot survey in a developing area, Cameroon. The Journal of tropical medicine and hygiene. 1992;95(1):57-61.

87. Nisingizwe MP, Makuza JD, Janjua NZ, Bansback N, Hedt-Gauthier B, Serumondo J, et al. The Cascade of Care for Hepatitis C Treatment in Rwanda: A Retrospective Cohort Study of the 2017-2019 Mass Screening and Treatment Campaign. Viruses. 2023;15(3).

88. Njoku OS, Manak MM, O'Connell RJ, Shutt ALW, Malia JA, Heipertz RA, Jr., et al. An Evaluation of Selected Populations for HIV-1 Vaccine Cohort Development in Nigeria. PLOS ONE. 2016;11(12):e0166711.

89. Njouom R, Caron M, Besson G, Ndong-Atome G-R, Makuwa M, Pouillot R, et al. Phylogeography, risk factors and genetic history of hepatitis C virus in Gabon, central Africa. PLOS ONE. 2012;7(8):e42002.

90. Njouom R, Pasquier C, Ayouba A, Gessain A, Froment A, Mfoupouendoun J, et al. High rate of hepatitis C virus infection and predominance of genotype 4 among elderly inhabitants of a remote village of the rain forest of South Cameroon. JOURNAL OF MEDICAL VIROLOGY. 2003;71(2):219-25.

91. Njouom R, Tejiokem MC, Texier G, Fontanet A. Prevalence of hepatitis B, hepatitis C and hepatitis D virus infections in Cameroon: Results from a national population based survey (The ANRS 12289 project). JOURNAL OF VIRAL HEPATITIS. 2015;22:1-2.

92. Nnadozie P, Okonko I, Nnadozie S, Hinderaker S. Serosurvey, awareness and sensitization on HIV, HBV, HCV and Mycobacterium tuberculosis among community-based population in Mbano, Imo State, Nigeria. Tropical Medicine and International Health. 2021;26:31-2.

93. Ntagirabiri R, Baransaka E, Ndayiragije A, Niyongabo T. Prevalence of hepatitis C virus in Burundi: A nationwide survey. Journal Africain d'Hepato-Gastroenterologie. 2014;8(1):25-8.

94. Nwankiti OO, Ndako JA, Echeonwu GO, Olabode AO, Nwosuh CI, Onovoh EM, et al. Hepatitis C Virus infection in apparentenly healthy individuals with family history of diabetes in Vom, Plateau State Nigeria. VIROLOGY JOURNAL. 2009;6:110.

95. O'Hara G, Mokaya J, Hau JP, Downs LO, McNaughton AL, Karabarinde A, et al. Liver function tests and fibrosis scores in a rural population in Africa: a cross-sectional study to estimate the burden of disease and associated risk factors. BMJ OPEN. 2020;10(3):e032890.

96. Odukoya OO, Odeyemi KA, Odubanjo OM, Isikekpei BC, Igwilo UU, Disu YM, et al. Hepatitis B and C seroprevalence among residents in Lagos State, Nigeria: A population-based survey. The Nigerian postgraduate medical journal. 2022;29(2):75-81.

97. Ogefere HO, Moses-Otutu IM, Igiezeme CI. Potential risk factors and seroprevalence of hepatitis c virus infection among students in a tertiary institution in Southern Nigeria. Journal of Medicine and Biomedical Research. 2016;15(2):5-11.

98. Okonkwo UC, Okpara H, Otu A, Ameh S, Ogarekpe Y, Osim H, et al. Prevalence of hepatitis B, hepatitis C and human immunodeficiency viruses, and evaluation of risk factors for transmission: Report of a population screening in Nigeria. SAMJ SOUTH AFRICAN MEDICAL JOURNAL. 2017;107(4):346-51.

99. Olal SO, Akere A, Otegbayo JA, Odaibo GN, Olaleye DO, Afolabi NB, et al. Are patients with primary hepatocellular carcinoma infectious of hepatitis B, C and D viruses? African journal of medicine and medical sciences. 2012;41:187-91.

100. Olalekan AW. Prevalence and risk factors for hepatitis B and C among sexually active undergraduates in southwestern Nigeria. ANNALS OF TROPICAL MEDICINE AND PUBLIC HEALTH. 2015;8(6):235-40.

101. Omer RE, Van't Veer P, Kadaru AM, Kampman E, el Khidir IM, Fedail SS, et al. The role of hepatitis B and hepatitis C viral infections in the incidence of hepatocellular carcinoma in Sudan. TRANSACTIONS OF THE ROYAL SOCIETY OF TROPICAL MEDICINE AND HYGIENE. 2001;95(5):487-91.

102. Omolade O, Adeyemi A. Prevalence of hepatitis C virus antibody among university students in Nigeria. JOURNAL OF VIRUS ERADICATION. 2018;4(4):228-9.

103. Onyekwere CA, Hameed L. Hepatitis B and C virus prevalence and association with demographics: report of population screening in Nigeria. TROPICAL DOCTOR. 2015;45(4):231-5.

104. Ouedraogo A, Kabore M, Barry A, Coulibaly S, Ouattara D, Kargougou DW, et al. Prevalence of HIV, hepatitis B and hepatitis C infection among potential participants to experimental vaccines trials in a rural area of Burkina Faso. AMERICAN JOURNAL OF TROPICAL MEDICINE AND HYGIENE. 2018;99(4):345.

105. Ouwe-Missi-Oukem-Boyer O, Ndouo FST, Ollomo B, Mezui-Me-Ndong J, Noulin F, Lachard I, et al. Hepatitis C virus infection may lead to slower emergence of P. falciparum in blood. PLOS ONE. 2011;6(1):e16034.

106. Owono MA, Roca ANS, Eburi E, Momo JC, Urbano V, Mochomuemue F, et al. Screening failures: How to improve health promotion and coverage of vaccination? AMERICAN JOURNAL OF TROPICAL MEDICINE AND HYGIENE. 2019;101(5):529.

107. Parr JB, Lodge E, Holzmayer V, Pepin J, Frost EH, Fried MW, et al. Dried blood spots allow for efficient, largescale surveys of hepatitis c viremia. AMERICAN JOURNAL OF TROPICAL MEDICINE AND HYGIENE. 2017;97(5):407.

108. Pennap GR, Yakubu A, Oyige O, Forbi J. Prevalence of hepatitis B and C virus infection among people of a local community in Keffi, Nigeria. AFRICAN JOURNAL OF MICROBIOLOGY RESEARCH. 2010;4(4):274-8.

109. Pepin J, Labbe A-C, Mamadou-Yaya F, Mbelesso P, Mbadingai S, Deslandes S, et al. Iatrogenic transmission of human T cell lymphotropic virus type 1 and hepatitis C virus through parenteral treatment and chemoprophylaxis of sleeping sickness in colonial Equatorial Africa. Clinical infectious diseases : an official publication of the Infectious Diseases Society of America. 2010;51(7):777-84.

110. Peto TJ, Mendy ME, Lowe Y, Webb EL, Whittle HC, Hall AJ. Efficacy and effectiveness of infant vaccination against chronic hepatitis B in the Gambia Hepatitis Intervention Study (1986-90) and in the nationwide immunisation program. BMC INFECTIOUS DISEASES. 2014;14:7.

111. Plamondon M, Labbe A-C, Frost E, Deslandes S, Alves AC, Bastien N, et al. Hepatitis C virus infection in Guinea-Bissau: a sexually transmitted genotype 2 with parenteral amplification? PLOS ONE. 2007;2(4):e372.

112. Puato M, Migliorato I, Tirrito C, Ruvoletto M, Zanardo M, Pauletto P, et al. Does HCV infection have a more favourable outcome in Tanzanian people?. Data from the Lugalawa study. DIGESTIVE AND LIVER DISEASE. 2007;39(9):891-2.

113. Quesada P, Whitby D, Benavente Y, Miley W, Labo N, Chichareon S, et al. Hepatitis C virus seroprevalence in the general female population from 8 countries. Journal of clinical virology : the official publication of the Pan American Society for Clinical Virology. 2015;68:89-93.

114. Ramarokoto CE, Rakotomanana F, Ratsitorahina M, Raharimanga V, Razafindratsimandresy R, Randremanana R, et al. Seroprevalence of hepatitis C and associated risk factors in urban areas of Antananarivo, Madagascar. BMC INFECTIOUS DISEASES. 2008;8:25.

115. Ramyil SC, Nimzing L, Lar N, Jonah PY, Dafam DD, Shik LP, et al. Prevalence and coinfection of human immunodeficiency virus, hepatitis B virus and hepatitis c virus in mabudi rural community of Plateau, State, Nigeria. SEXUALLY TRANSMITTED DISEASES. 2014;41:S144.

116. Richard-Lenoble D, Traore O, Kombila M, Roingeard P, Dubois F, Goudeau A. Hepatitis B, C, D, and E markers in rural equatorial African villages (Gabon). The American journal of tropical medicine and hygiene. 1995;53(4):338-41.

117. Rodgers M, Holzmayer V, Vallari A, Olivo A, Forberg K, Fuhrman J, et al. Hepatitis C virus and human pegivirus 2 surveillance in a large cameroonian cohort. Topics in Antiviral Medicine. 2018;26:271s.

118. Sentjens RE, Sisay Y, Vrielink H, Kebede D, Reesink HW. Risk factors for HCV, HTLV-I/II and HIV infection in various population subsets in Ethiopia. TRANSFUSION. 2000;40(10):80S-S.

119. Siawaya JFD, Kouegnigan Rerambiah L. HIV, HBS, HCV, HTLV and syphilis prevalence among high school learners and university student in Libreville, Gabon. VULNERABLE CHILDREN AND YOUTH STUDIES. 2015;10(3):220-4.

120. Stark K, Poggensee G, Hohne M, Bienzle U, Kiwelu I, Schreier E. Seroepidemiology of TT virus, GBC-C/HGV, and hepatitis viruses B, C, and E among women in a rural area of Tanzania. JOURNAL OF MEDICAL VIROLOGY. 2000;62(4):524-30.

121. Stevens W, Kamali A, Karita E, Anzala O, Sanders EJ, Jaoko W, et al. Baseline morbidity in 2,990 adult African volunteers recruited to characterize laboratory reference intervals for future HIV vaccine clinical trials. PLOS ONE. 2008;3(4):e2043.

122. Stockdale AJ, Kreuels B, Shawa IT, Meiring JE, Thindwa D, Silungwe NM, et al. A clinical and molecular epidemiological survey of hepatitis C in Blantyre, Malawi, suggests a historic mechanism of transmission. JOURNAL OF VIRAL HEPATITIS. 2022;29(4):252-62.

123. Sube KL, Seriano O, Lako J, Ochero C, Lasuba A, Lino E, et al. Prevalence of HBV, HCV, HIV and syphilis infections among secondary school students in Juba, South Sudan. AMERICAN JOURNAL OF TROPICAL MEDICINE AND HYGIENE. 2020;103(5):9-10.

124. Taha TE, Rusie LK, Labrique A, Nyirenda M, Soko D, Kamanga M, et al. Seroprevalence for Hepatitis E and Other Viral Hepatitides among Diverse Populations, Malawi. EMERGING INFECTIOUS DISEASES. 2015;21(7):1174-82.

125. Tao I, Compaore TR, Diarra B, Djigma F, Zohoncon TM, Assih M, et al. Seroepidemiology of hepatitis B and C viruses in the general population of Burkina Faso. Hepatitis Research and Treatment. 2014;2014:781843.

126. Taye BD, Kassa T, Teshager L, Kedir R, Yeshanew AG, Aseffa A, et al. Prevalence and associated risk factors of Hepatitis B and C virus infections among mothers in Jimma, South West Ethiopia: a community-based study. Ethiop med j (Online). 2019;57(3):109-17.

127. Tazinkeng NN, Teuwafeu DG, Asombang AW, Ndip VA, Bloom SM, Nkhoma A, et al. Prevalence of and factors associated with hepatitis b and c in an urban health district in Cameroon, West Africa: A cross-sectional study. AMERICAN JOURNAL OF GASTROENTEROLOGY. 2021;116:S520.

128. Tess BH, Levin A, Brubaker G, Shao J, Drummond JE, Alter HJ, et al. Seroprevalence of hepatitis C virus in the general population of northwest Tanzania. The American journal of tropical medicine and hygiene. 2000;62(1):138-41.

129. Ugah UI, Alo MN, Gloria UC. Epidemiology of hepatitis b virus, hepatitis C virus and human immunodeficiency virus co-infection among assymptomatic persons resident in Alex Ekwueme federal university Ndufu-alike. SCIENTIFIC AFRICAN. 2021;14.

130. Umutesi J, Liu CY, Penkunas MJ, Makuza JD, Ntihabose CK, Umuraza S, et al. Screening a nation for hepatitis C virus elimination: a cross-sectional study on prevalence of hepatitis C and associated risk factors in the Rwandan general population. BMJ OPEN. 2019;9(7):e029743.

131. Woldegiorgis AE, Erku W, Medhin G, Berhe N, Legesse M. Community-based sero-prevalence of hepatitis B and C infections in South Omo Zone, Southern Ethiopia. PLOS ONE. 2019;14(12):e0226890.

132. Zeller H, Rabarijaona L, Rakoto-Andrianarivelo M, Boisier P. [Prevalence of hepatitis C virus infection in the general population of Madagascar]. Prevalence de l'infection par le virus de l'hepatite C en population generale a Madagascar. 1997;90(1):3-5.

133. Besombes C, Njouom R, Paireau J, Lachenal G, Texier G, Tejiokem M, et al. The epidemiology of hepatitis delta virus infection in Cameroon. GUT. 2020;69(7):1294-300.

134. Kpossou AR, Paraiso MN, Sokpon CN, Alassan KS, Vignon RK, Keke RK, et al. Viral hepatitis B during a general population screening campaign in Benin: seroprevalence and associated factors. PAN AFRICAN MEDICAL JOURNAL. 2020;37.

135. Mudawi H, Smith H, Rohud S, Saeed O, Fedail S. Prevalence of HCV infection in an endemic area for schistosomal infection and in patients with hepatosplenic schistosomiasis in Sudan. LIVER INTERNATIONAL. 2006;26:75-.

136. Ndow G, Cessay A, Cohen D, Shimakawa Y, Gore ML, Tamba S, et al. Prevalence and Clinical Significance of Occult Hepatitis B Infection in The Gambia, West Africa. The Journal of infectious diseases. 2022;226(5):862-70.

137. Njouom R, Siffert I, Texier G, Lachenal G, Tejiokem MC, Pepin J, et al. The burden of hepatitis C virus in Cameroon: Spatial epidemiology and historical perspective. JOURNAL OF VIRAL HEPATITIS. 2018;25(8):959-68.

138. Parr JB, Lodge EK, Holzmayer V, Pepin J, Frost EH, Fried MW, et al. An Efficient, Large-Scale Survey of Hepatitis C Viremia in the Democratic Republic of the Congo Using Dried Blood Spots. Clinical infectious diseases : an official publication of the Infectious Diseases Society of America. 2018;66(2):254-60.

139. Pepin J, Lavoie M, Pybus OG, Pouillot R, Foupouapouognigni Y, Rousset D, et al. Risk factors for hepatitis C virus transmission in colonial Cameroon. Clinical infectious diseases : an official publication of the Infectious Diseases Society of America. 2010;51(7):768-76.

140. Razafindratsimandresy R, Dubot A, Ramarokoto C-E, Iehle C, Soares J-L, Rousset D. Hepatitis C virus infection and genotypes in Antananarivo, Madagascar. JOURNAL OF MEDICAL VIROLOGY. 2007;79(8):1082-8.

141. Tazinkeng NN, Teuwafeu DG, Asombang AW, Agbor VN, Bloom SM, Nkhoma AN, et al. Factors associated with hepatitis B and C among adults in Buea, Cameroon: A community-based cross-sectional study. Liver international : official journal of the International Association for the Study of the Liver. 2022;42(11):2396-402.
